# Supplementary material for: Koina: Democratizing machine learning for proteomics research
Source: Nat Commun. 2025 Nov 11;16:9933. doi: 10.1038/s41467-025-64870-5 (PMC12606132; doi:10.1038/s41467-025-64870-5)
Supplement: Supplementary file 1 — Supplementary Information [file 41467_2025_64870_MOESM1_ESM.pdf]

Supplementary Information

Koina: Democratizing machine learning for proteomics research

Authors
Ludwig Lautenbacher 1,2,#; Kevin L. Yang 3,#; Tobias Kockmann 4; Christian Panse 4,5; Wassim Gabriel 1; Dulguun Bold 1; Elias Kahl 1; Matthew Chambers 6; Brendan X. MacLean 6; Kai Li 3; Fengchao Yu 7; Brian C. Searle 8,9,10; Wilburn, Damien 10; Mohammad Reza Zare Shahneh 11; Yuhui Hong 12; Haixu Tang 12; Mingxun Wang 11,13; Ralf Gabriels 14,15; Robbin Bouwmeester 14,15; Robbe Devreese 14,15; Jesse Angelis 1; Eduard Sabidó 16,17; Tobias K. Schmidt 18; Alexey I. Nesvizhskii 3,7,*; Mathias Wilhelm 1,2,*

# Contributed equally

Corresponding authors (*)
Alexey I. Nesvizhskii, nesvi@med.umich.edu
Mathias Wilhelm, mathias.wilhelm@tum.de

Affiliations

1. Computational Mass Spectrometry, Technical University of Munich (TUM), Freising, Germany
2. Munich Data Science Institute, Technical University of Munich, 85748, Garching, Germany
3. Gilbert S. Omenn Department of Computational Medicine and Bioinformatics, University of Michigan, Ann Arbor, MI, USA
4. Functional Genomics Center Zurich (FGCZ) - University of Zurich | ETH Zurich, Winterthurerstrasse 190, CH-8057 Zurich, Switzerland
5. Swiss Institute of Bioinformatics (SIB), Quartier Sorge - Batiment Amphipole, CH-1015 Lausanne, Switzerland
6. Department of Genome Sciences, University of Washington, Seattle, WA 98195
7. Department of Pathology, University of Michigan, Ann Arbor, MI, USA
8. Pelotonia Institute for Immuno-Oncology, The Ohio State University Comprehensive Cancer Center, Columbus, Ohio
9. Department of Biomedical Informatics, The Ohio State University, Columbus, Ohio
10. Department of Chemistry and Biochemistry, The Ohio State University, Columbus, Ohio
11. Department of Computer Science, University of California Riverside, Riverside, CA, USA
12. Luddy School of Informatics, Computing, and Engineering, Indiana University Bloomington, Bloomington, IN 47408, United States
13. Virtual Multi-Omics Laboratory, The Internet, Riverside, CA, USA

14. VIB-UGent Center for Medical Biotechnology, VIB, 9052 Ghent, Belgium
15. Department of Biomolecular Medicine, Ghent University, 9052 Ghent, Belgium
16. Centre for Genomic Regulation (CRG), The Barcelona Institute of Science and Technology (BIST), Dr. Aiguader 88, Barcelona 08003, Spain
17. Universitat Pompeu Fabra (UPF), Dr. Aiguader 88, Barcelona 08003, Spain
18. MSAID GmbH, Garching, Germany

Supplementary Information 1
Supplementary Figure 1 4
Supplementary Figure 2 5
Supplementary Figure 3 5
Supplementary Figure 4 6
Supplementary Figure 5 7
Supplementary Figure 6 8
Supplementary Figure 7 10
Supplementary Figure 8 12
Supplementary Figure 9 14
Supplementary Figure 10 16
Supplementary Figure 11 18
Supplementary Figure 12 20
Supplementary Figure 13 22
Supplementary Figure 14 24
Supplementary Figure 15 24
Supplementary Figure 16 26
Supplementary Figure 17 28
Supplementary Figure 18 30
Supplementary Figure 19 31
Supplementary Figure 20 31
Supplementary Figure 21 35
Supplementary Figure 22 38
Supplementary Table 1 39
Comparison with existing platforms 39
BioModelsML 39
Kipoi 39
Additional MSBooster PSM rescoring 40
Introduction 40
Phosphoproteomics 40
Astral DIA 41
TMT 42
Tutorials 44
References 44

Supplementary Figures

a

```python
from koinapy import Koina
import numpy as np
import pandas as pd

inputs = pd.DataFrame()
inputs['peptide_sequences'] = np.array(["AAAAAKAK", "AAAAAKAK"])
inputs['precursor_charges'] = np.array([1,2])
inputs['collision_energies'] = np.array([25, 25])

model = Koina("Prosit_2019_intensity", "koina.wilhelmlab.org:443")
predictions = model.predict(inputs)
```

b

peptide_sequences	precursor_charges	collision_energies	mz	intensities	annotation
AAAAAKAK	1	25	147.112808	0.461771	b'y1+1'
AAAAAKAK	1	25	72.044388	0.068091	b'b1+1'
AAAAAKAK	1	25	218.149918	0.328826	b'y2+1'
AAAAAKAK	1	25	143.081497	0.136333	b'b2+1'
AAAAAKAK	1	25	346.244873	0.606697	b'y3+1'
AAAAAKAK	1	25	214.118622	0.476283	b'b3+1'
AAAAAKAK	1	25	417.281982	0.825222	b'y4+1'
AAAAAKAK	1	25	285.155731	0.628604	b'b4+1'
AAAAAKAK	1	25	488.319122	0.800198	b'y5+1'
AAAAAKAK	1	25	356.192841	0.667753	b'b5+1'
AAAAAKAK	1	25	559.356201	0.666764	b'y6+1'
AAAAAKAK	1	25	484.287811	1.000000	b'b6+1'
AAAAAKAK	1	25	630.393311	0.240440	b'y7+1'
AAAAAKAK	1	25	555.324951	0.871939	b'b7+1'

Supplementary Figure 1

(a) Code example for calling the Prosit_2019_intensity model using the KoinaPy client library as seen on koina.wilhelmlab.org. (b) Example output as generated by a call using KoinaPy.

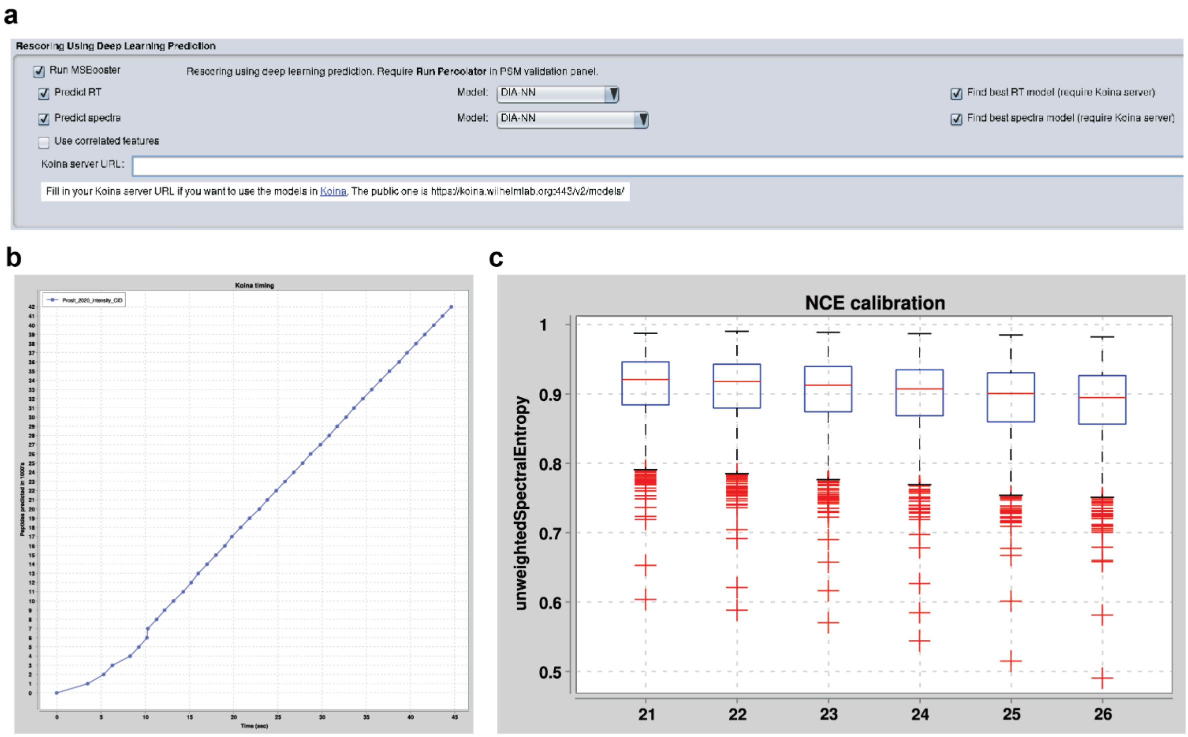

Supplementary Figure 2

(a) Screenshot of the MSBooster section under the FragPipe Validation tab. RT and spectral features can be used together or separately, with a dropdown menu to choose a model. If “Find best RT/spectra model” is checked, then a heuristic algorithm is enabled to attempt to determine the combination of models that maximizes peptides identified. One can also manually set an RT model but enable search for the best spectra model, and vice versa. A textbox is included to set the path to the Koina server, which may be a private, locally hosted one, or the public one provided. (b) Peptides are submitted in batches of 1000 to the Koina server. Total time taken is recorded on the x-axis. (c) Similarity box-and-whisker plot across sequential normalized collision energy (NCE) values for the PSMs collection during the NCE calibration step. The top whisker is the third quartile plus 1.5 times the interquartile range.

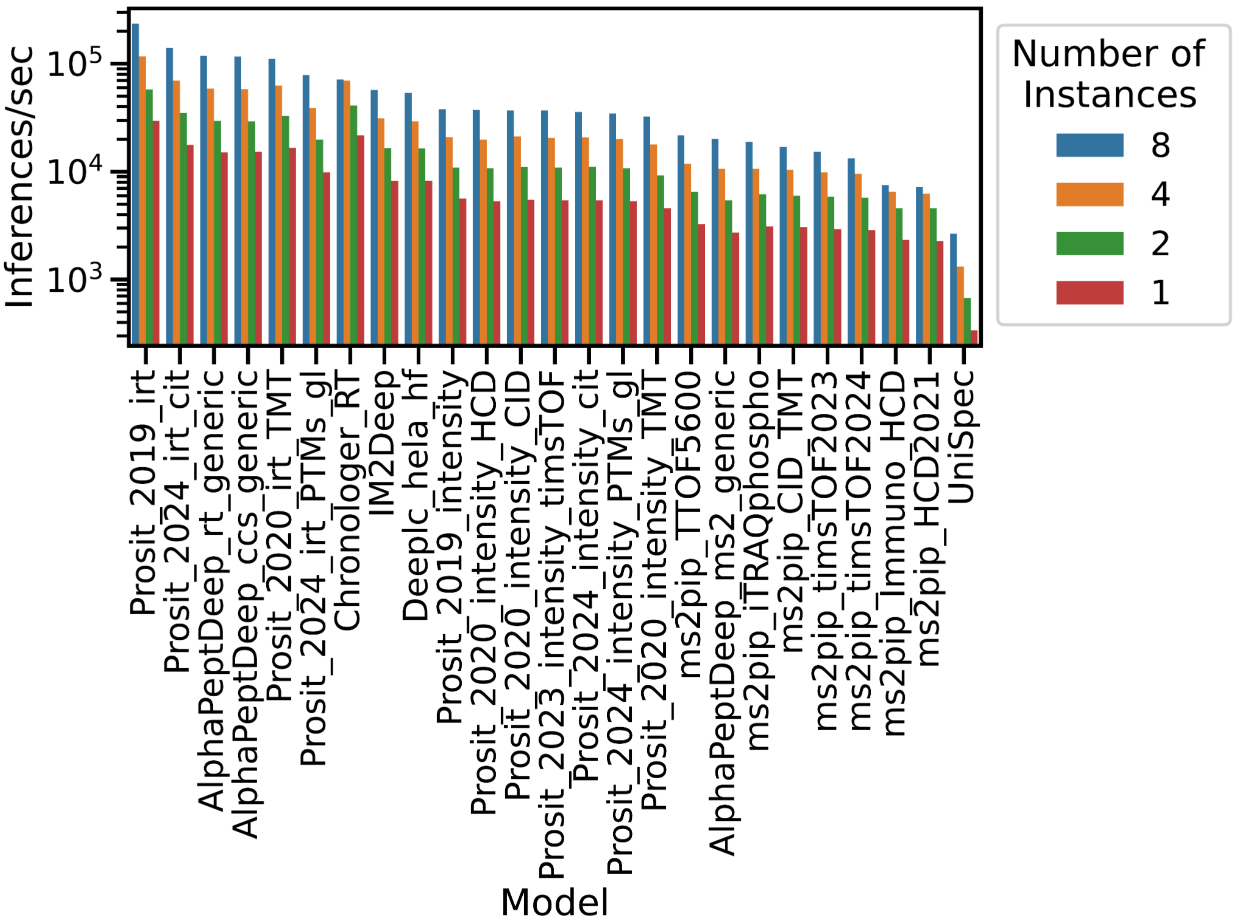

Supplementary Figure 3

Benchmarking results of models available on Koina measured in Inferences per second according to model and Number of Koina instances used. The client location is Freising, Germany server location is Freising, Germany. All peptides used in this Benchmark have a length of 15 amino acids. Source data are provided as a Source Data file.

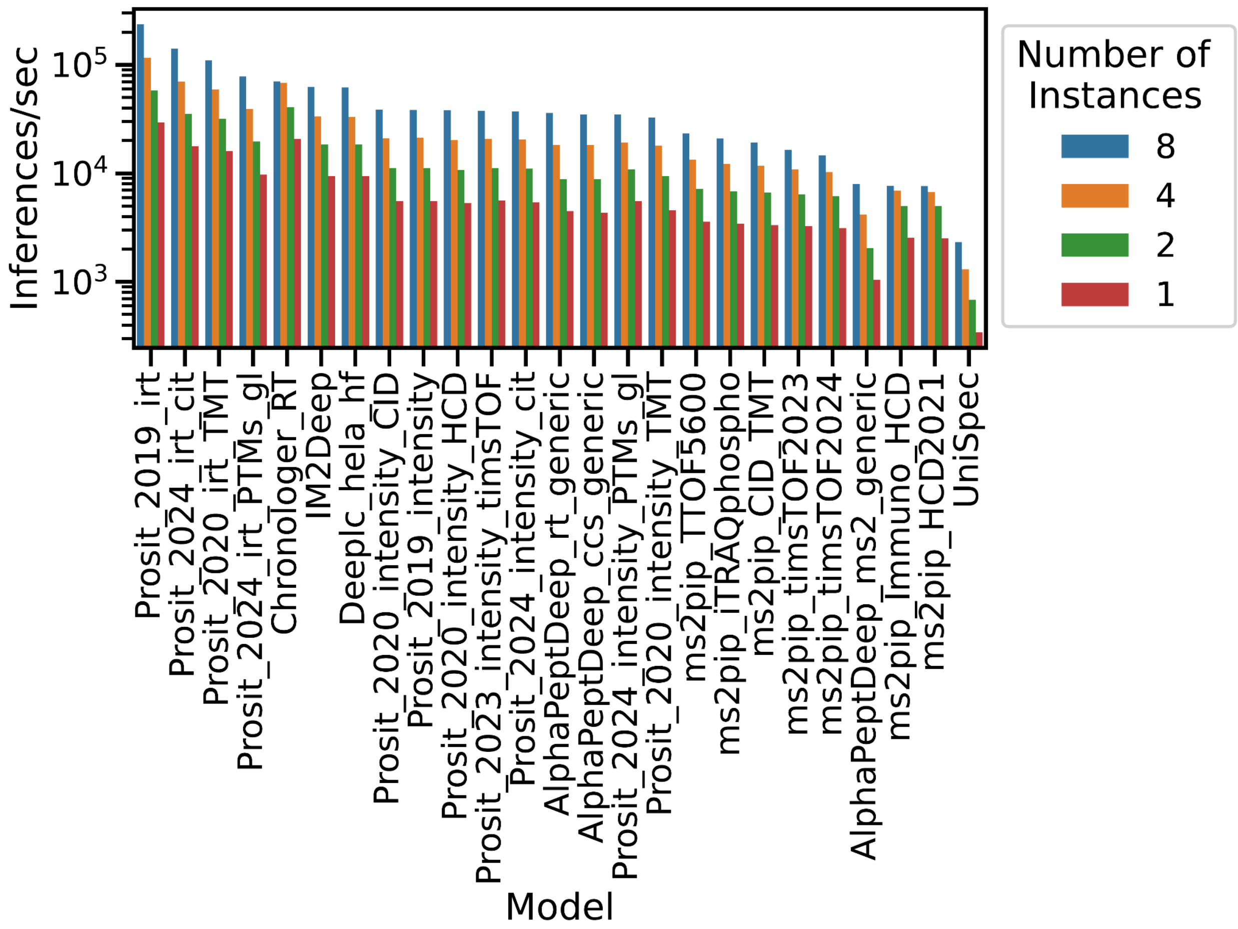

Supplementary Figure 4

Benchmarking results of models available on Koina are measured in inferences per second according to the model and the number of Koina instances used. The client location is Freising, Germany server location is Freising, Germany. Peptides used in this Benchmark have a length between 7 and 30 amino acids. Source data are provided as a Source Data file.

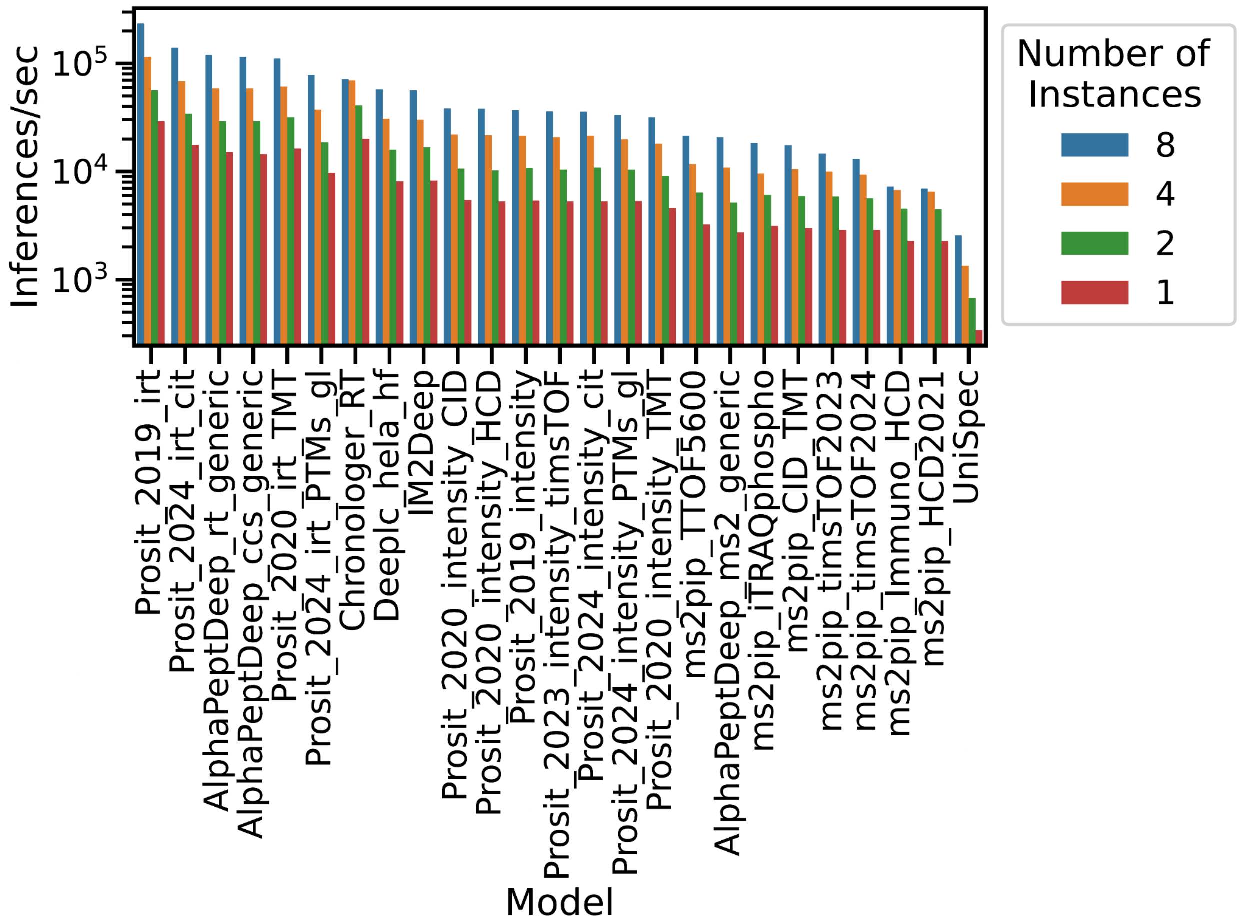

Supplementary Figure 5

Benchmarking results of models available on Koina measured in Inferences per second according to model and Number of Koina instances used. The client location is Ann Arbor, Michigan, USA server location is Freising, Germany. All peptides used in this Benchmark have a length of 15 amino acids. Source data are provided as a Source Data file.

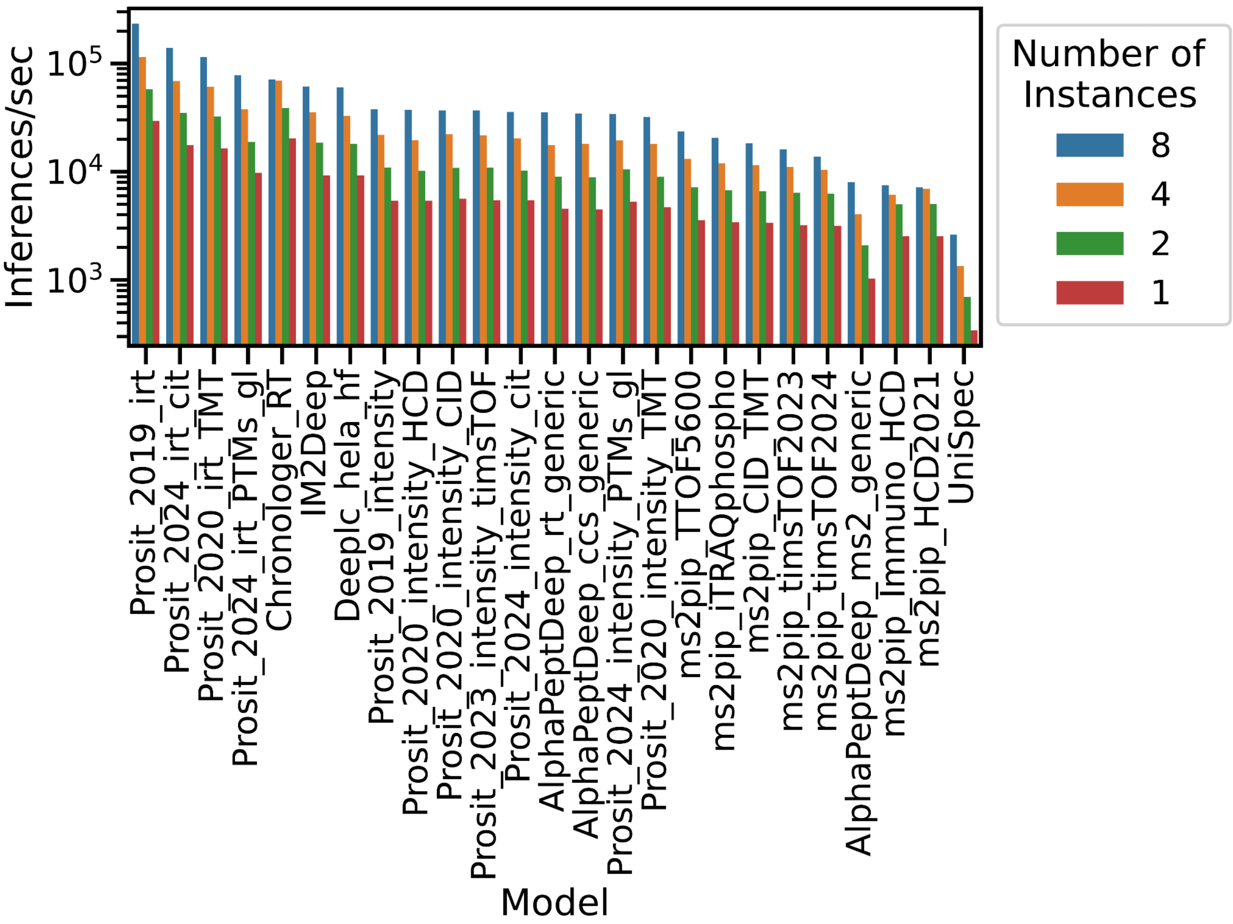

Supplementary Figure 6

Benchmarking results of models available on Koina measured in Inferences per second according to model and Number of Koina instances used. The client location is Ann Arbor, Michigan, USA server location is Freising, Germany. Peptides used in this Benchmark have a length between 7 and 30 amino acids. Source data are provided as a Source Data file.

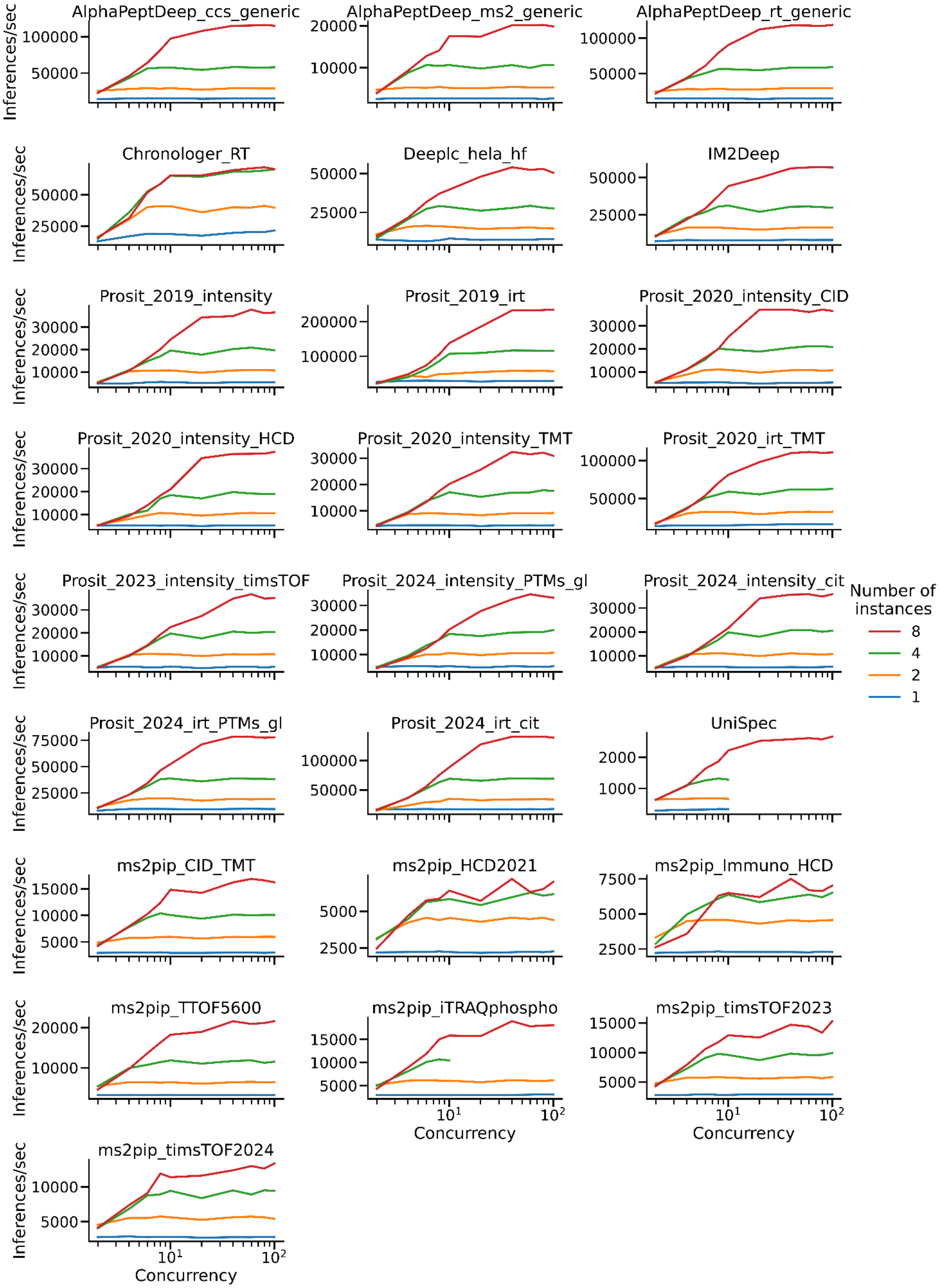
AlphaPeptDeep_ccs_generic
AlphaPeptDeep_ms2_generic
AlphaPeptDeep_rt_generic
Chronologer_RT
Deeplc_hela_hf
IM2Deep
Prosit_2019_intensity
Prosit_2019_irt
Prosit_2020_intensity_CID
Prosit_2020_intensity_HCD
Prosit_2020_intensity_TMT
Prosit_2020_irt_TMT
Prosit_2023_intensity_timsTOF
Prosit_2024_intensity_PTMs_gl
Prosit_2024_intensity_cit
Prosit_2024_irt_PTMs_gl
Prosit_2024_irt_cit
UniSpec
ms2pip_CID_TMT
ms2pip_HCD2021
ms2pip_Immuno_HCD
ms2pip_TTOF5600
ms2pip_iTRAQphospho
ms2pip_timsTOF2023
ms2pip_timsTOF2024
Inferences/sec
Concurrency
Number of instances
8
4
2
1

Supplementary Figure 7

Benchmarking results of models available on Koina measured in Inferences per second according to model, client-side concurrency (2,4,6,8,10,20,40,60,80,100) and Number of Koina instances used. The client location is Freising, Germany server location is Freising, Germany. Peptides used in this Benchmark have a length of 15 amino acids. Source data are provided as a Source Data file.

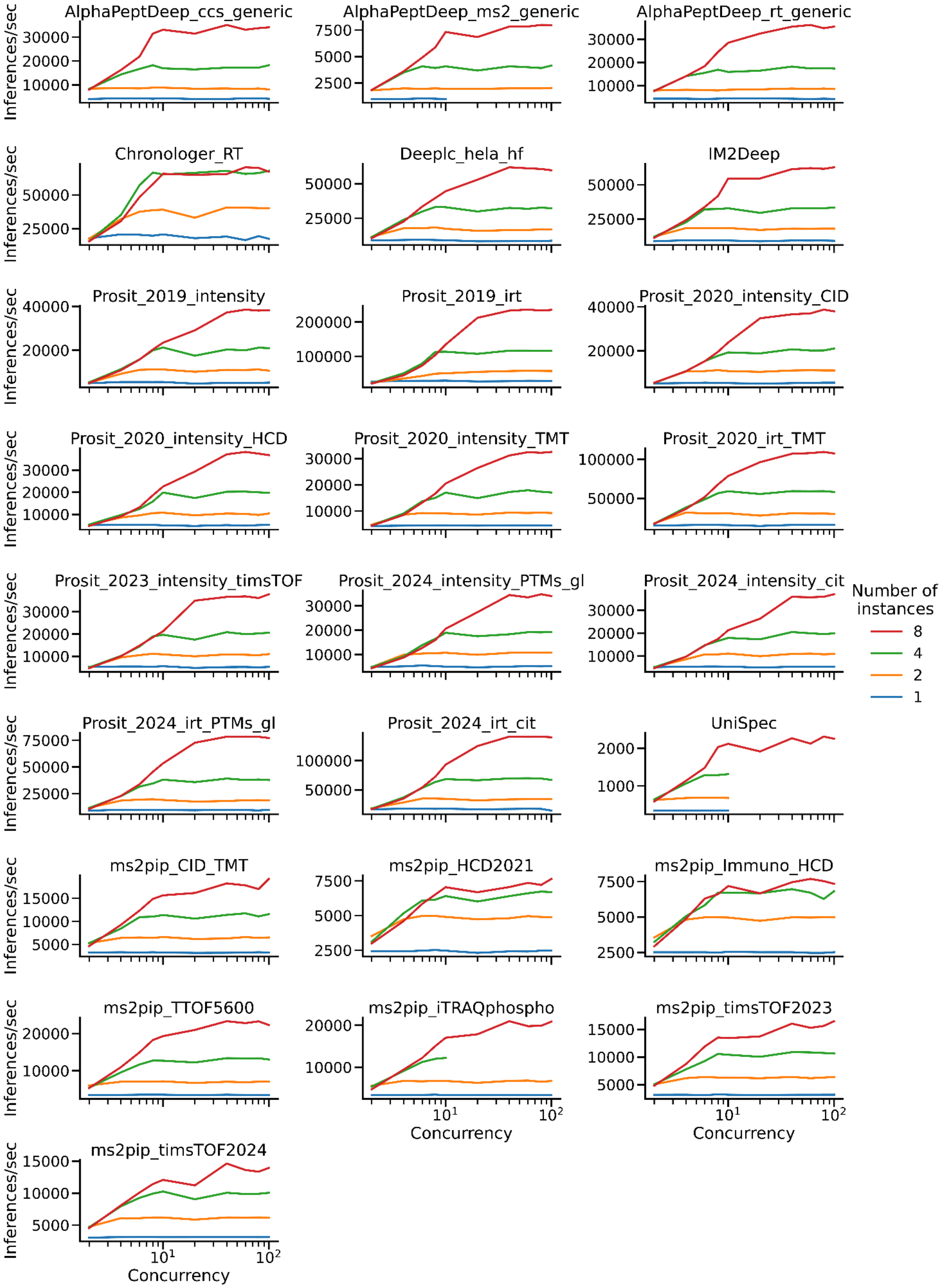
AlphaPeptDeep_ccs_generic
AlphaPeptDeep_ms2_generic
AlphaPeptDeep_rt_generic
Chronologer_RT
Deeplc_hela_hf
IM2Deep
Prosit_2019_intensity
Prosit_2019_irt
Prosit_2020_intensity_CID
Prosit_2020_intensity_HCD
Prosit_2020_intensity_TMT
Prosit_2020_irt_TMT
Prosit_2023_intensity_timsTOF
Prosit_2024_intensity_PTMs_gl
Prosit_2024_intensity_cit
Prosit_2024_irt_PTMs_gl
Prosit_2024_irt_cit
UniSpec
ms2pip_CID_TMT
ms2pip_HCD2021
ms2pip_Immuno_HCD
ms2pip_TTOF5600
ms2pip_iTRAQphospho
ms2pip_timsTOF2023
ms2pip_timsTOF2024
Inferences/sec
Concurrency
Number of instances
8
4
2
1

Supplementary Figure 8

Benchmarking results of models available on Koina measured in Inferences per second according to model, client-side concurrency (2,4,6,8,10,20,40,60,80,100) and Number of Koina instances used. The client location is Freising, Germany server location is Freising, Germany. Peptides used in this Benchmark have a length between 7 and 30 amino acids. Source data are provided as a Source Data file.

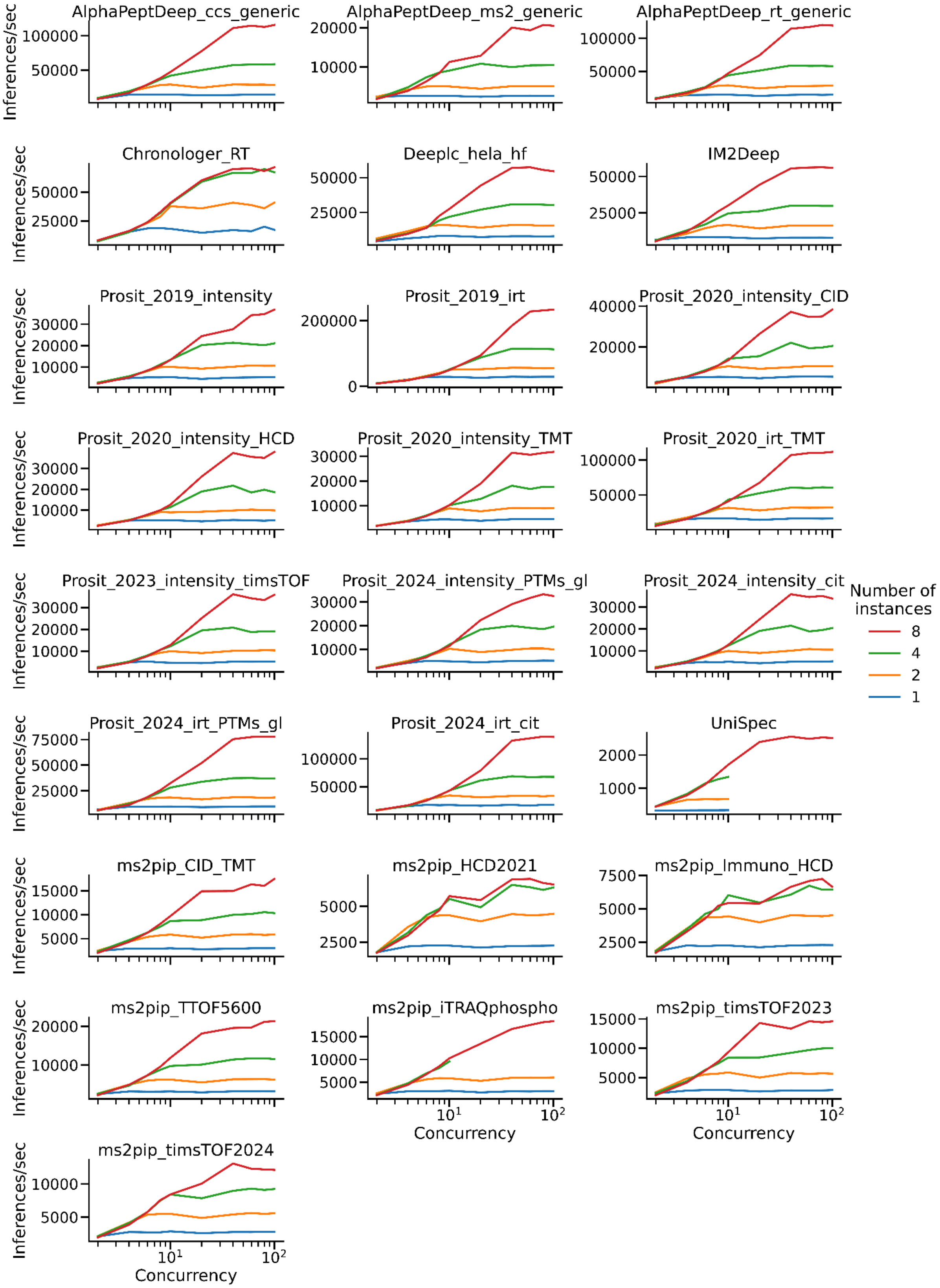
AlphaPeptDeep_ccs_generic
AlphaPeptDeep_ms2_generic
AlphaPeptDeep_rt_generic
Chronologer_RT
Deeplc_hela_hf
IM2Deep
Prosit_2019_intensity
Prosit_2019_irt
Prosit_2020_intensity_CID
Prosit_2020_intensity_HCD
Prosit_2020_intensity_TMT
Prosit_2020_irt_TMT
Prosit_2023_intensity_timsTOF
Prosit_2024_intensity_PTMs_gl
Prosit_2024_intensity_cit
Prosit_2024_irt_PTMs_gl
Prosit_2024_irt_cit
UniSpec
ms2pip_CID_TMT
ms2pip_HCD2021
ms2pip_Immuno_HCD
ms2pip_TTOF5600
ms2pip_iTRAQphospho
ms2pip_timsTOF2023
ms2pip_timsTOF2024
Inferences/sec
Concurrency
Number of instances
8
4
2
1

Supplementary Figure 9

Benchmarking results of models available on Koina measured in Inferences per second according to model, client-side concurrency (2,4,6,8,10,20,40,60,80,100) and Number of Koina instances used. The client location is Ann Arbor, Michigan, USA server location is Freising, Germany. Peptides used in this Benchmark have a length of 15 amino acids. Source data are provided as a Source Data file.

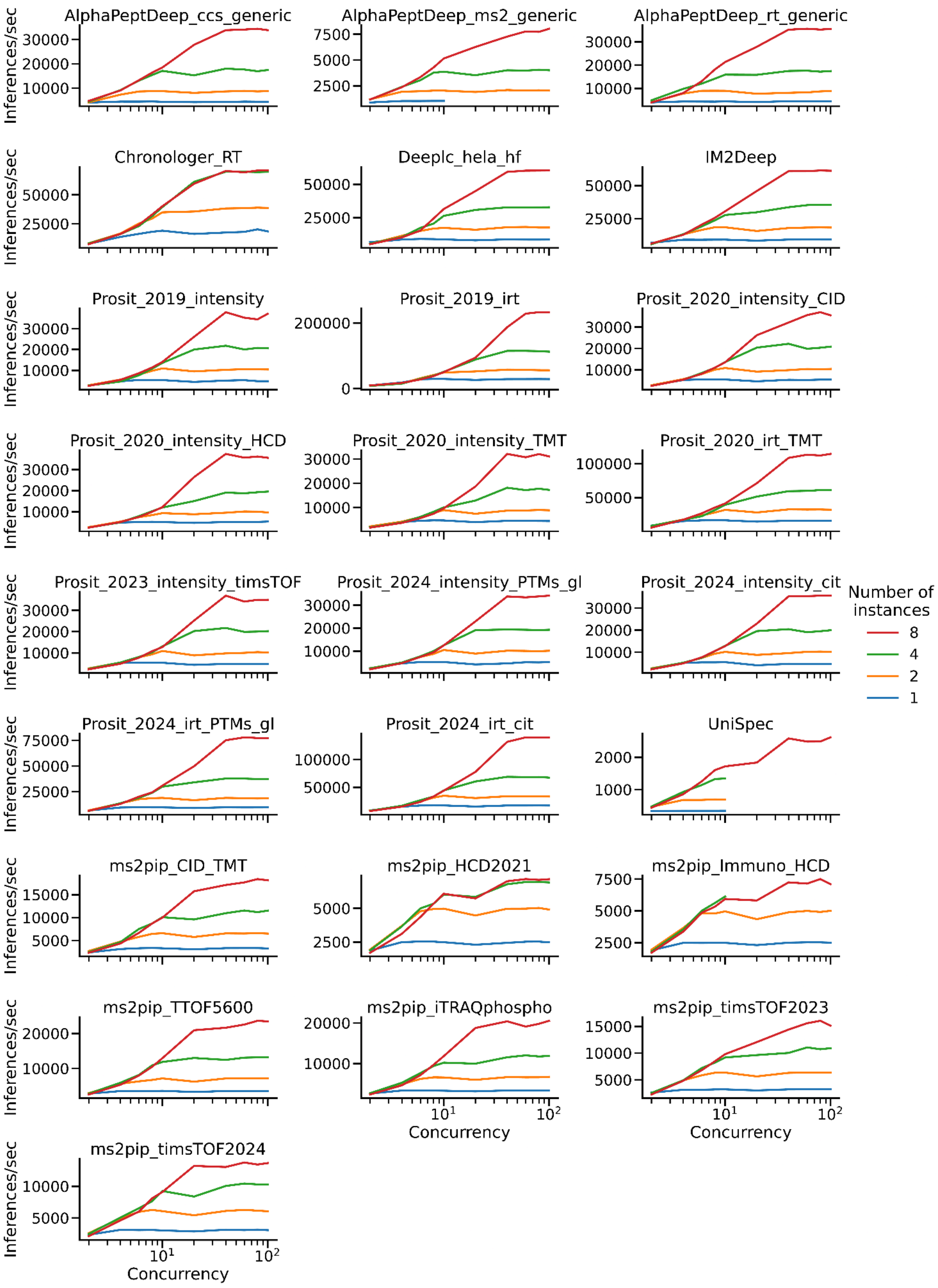
AlphaPeptDeep_ccs_generic
AlphaPeptDeep_ms2_generic
AlphaPeptDeep_rt_generic
Chronologer_RT
Deeplc_hela_hf
IM2Deep
Prosit_2019_intensity
Prosit_2019_irt
Prosit_2020_intensity_CID
Prosit_2020_intensity_HCD
Prosit_2020_intensity_TMT
Prosit_2020_irt_TMT
Prosit_2023_intensity_timsTOF
Prosit_2024_intensity_PTMs_gl
Prosit_2024_intensity_cit
Prosit_2024_irt_PTMs_gl
Prosit_2024_irt_cit
UniSpec
ms2pip_CID_TMT
ms2pip_HCD2021
ms2pip_Immuno_HCD
ms2pip_TTOF5600
ms2pip_iTRAQphospho
ms2pip_timsTOF2023
ms2pip_timsTOF2024
Inferences/sec
Concurrency
Number of instances
8
4
2
1

Supplementary Figure 10

Benchmarking results of models available on Koina measured in Inferences per second according to model, client-side concurrency (2,4,6,8,10,20,40,60,80,100) and Number of Koina instances used. The client location is Ann Arbor, Michigan, USA server location is Freising, Germany. Peptides used in this Benchmark have a length between 7 and 30 amino acids. Source data are provided as a Source Data file.

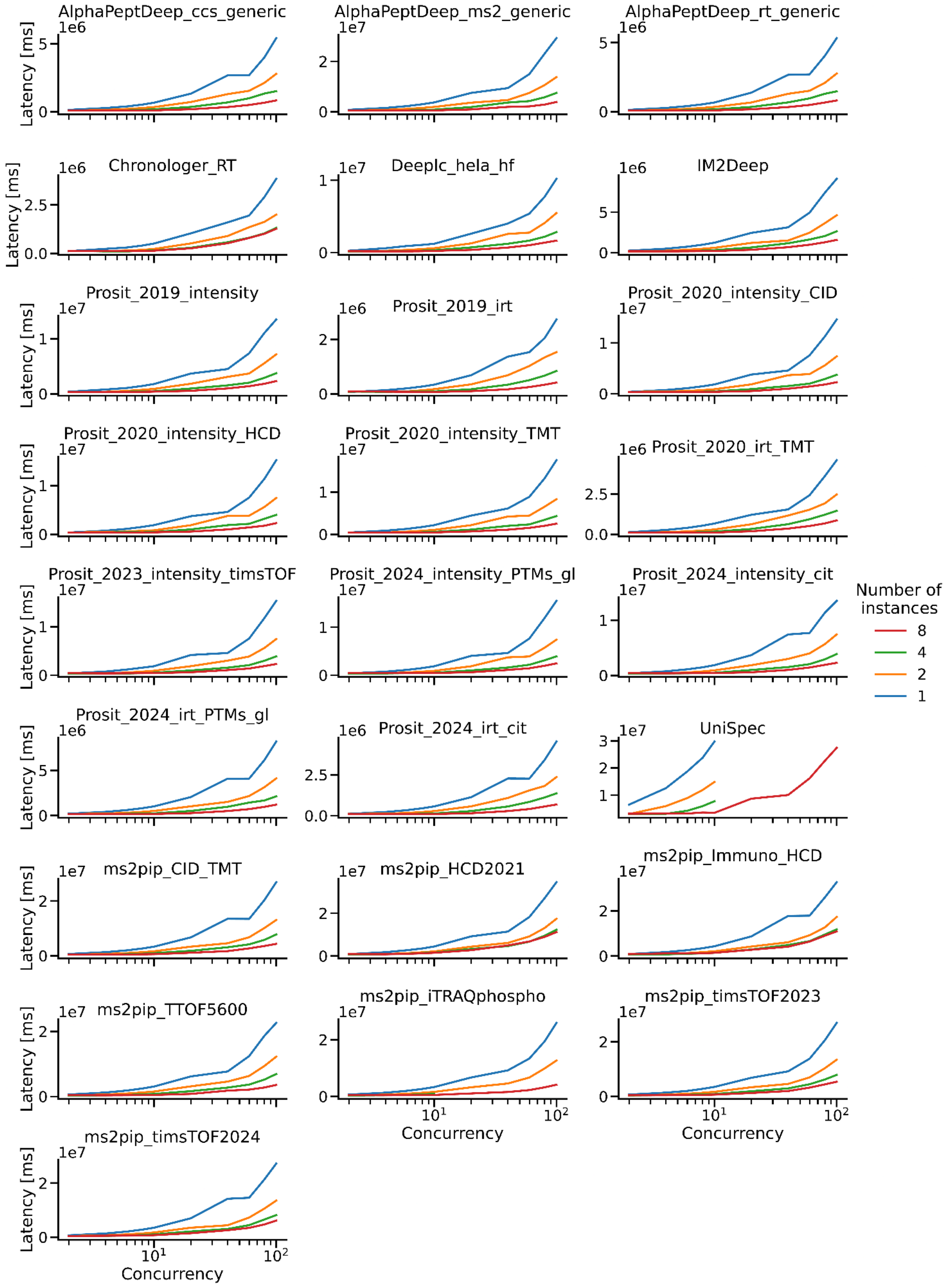
AlphaPeptDeep_ccs_generic
AlphaPeptDeep_ms2_generic
AlphaPeptDeep_rt_generic
Chronologer_RT
Deeplc_hela_hf
IM2Deep
Prosit_2019_intensity
Prosit_2019_irt
Prosit_2020_intensity_CID
Prosit_2020_intensity_HCD
Prosit_2020_intensity_TMT
Prosit_2020_irt_TMT
Prosit_2023_intensity_timsTOF
Prosit_2024_intensity_PTMs_gl
Prosit_2024_intensity_cit
Prosit_2024_irt_PTMs_gl
Prosit_2024_irt_cit
UniSpec
ms2pip_CID_TMT
ms2pip_HCD2021
ms2pip_Immuno_HCD
ms2pip_TTOF5600
ms2pip_iTRAQphospho
ms2pip_timsTOF2023
ms2pip_timsTOF2024
Latency [ms]
Concurrency
Number of instances
8
4
2
1

Supplementary Figure 11

Benchmarking results of models available on Koina measured in median latency in microseconds according to model, client-side concurrency (2,4,6,8,10,20,40,60,80,100), and number of Koina instances used. The client location is Freising, Germany server location is Freising, Germany. Peptides used in this Benchmark have a length of 15 amino acids. Source data are provided as a Source Data file.

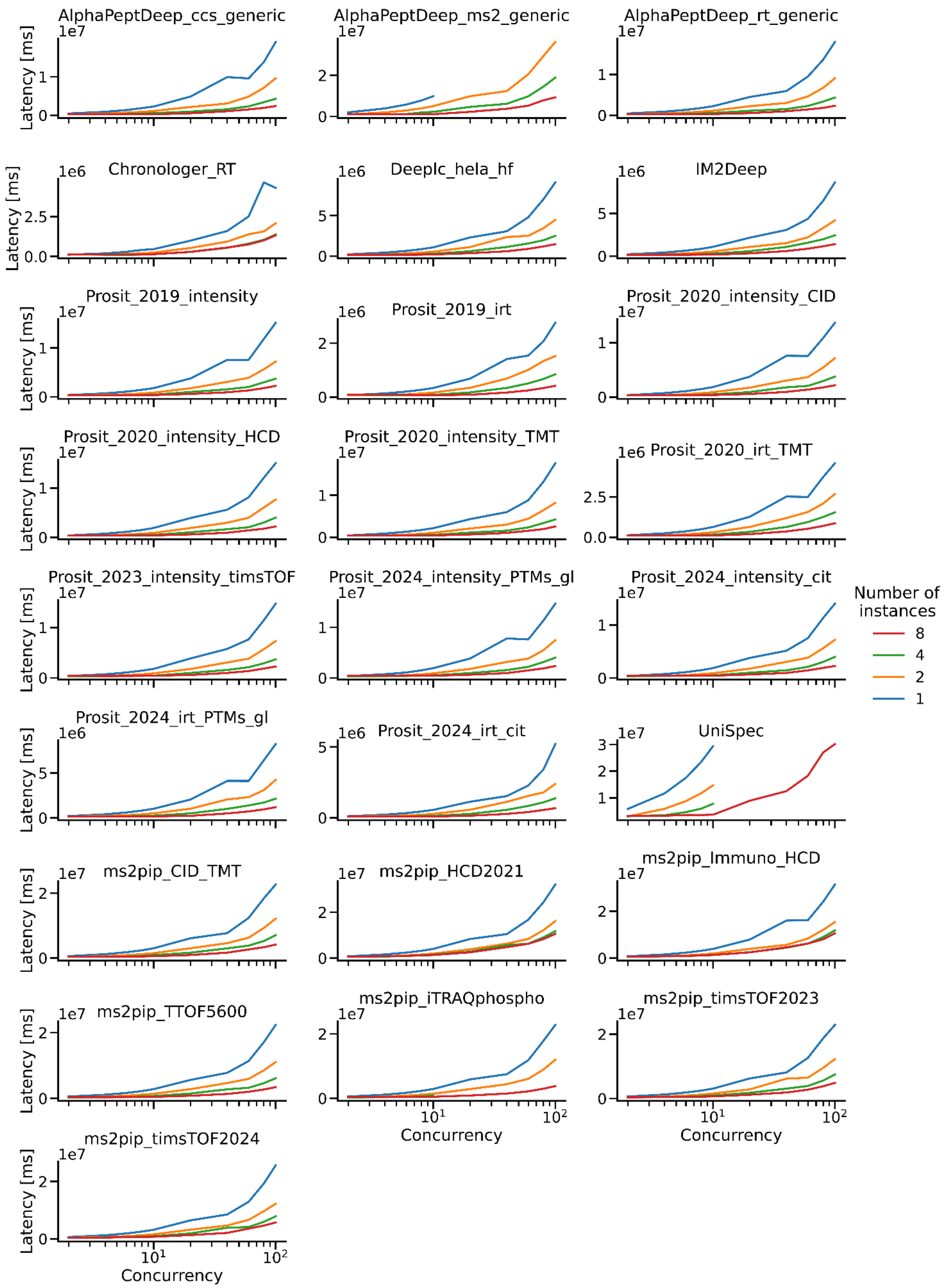
AlphaPeptDeep_ccs_generic
AlphaPeptDeep_ms2_generic
AlphaPeptDeep_rt_generic
Chronologer_RT
Deeplc_hela_hf
IM2Deep
Prosit_2019_intensity
Prosit_2019_irt
Prosit_2020_intensity_CID
Prosit_2020_intensity_HCD
Prosit_2020_intensity_TMT
Prosit_2020_irt_TMT
Prosit_2023_intensity_timsTOF
Prosit_2024_intensity_PTMs_gl
Prosit_2024_intensity_cit
Prosit_2024_irt_PTMs_gl
Prosit_2024_irt_cit
UniSpec
ms2pip_CID_TMT
ms2pip_HCD2021
ms2pip_Immuno_HCD
ms2pip_TTOF5600
ms2pip_iTRAQphospho
ms2pip_timsTOF2023
ms2pip_timsTOF2024
Latency [ms]
Concurrency
Number of instances
8
4
2
1

Supplementary Figure 12

Benchmarking results of models available on Koina measured in median latency in microseconds according to model, client-side concurrency (2,4,6,8,10,20,40,60,80,100), and number of Koina instances used. The client location is Freising, Germany server location is Freising, Germany. Peptides used in this Benchmark have a length between 7 and 30 amino acids. Source data are provided as a Source Data file.

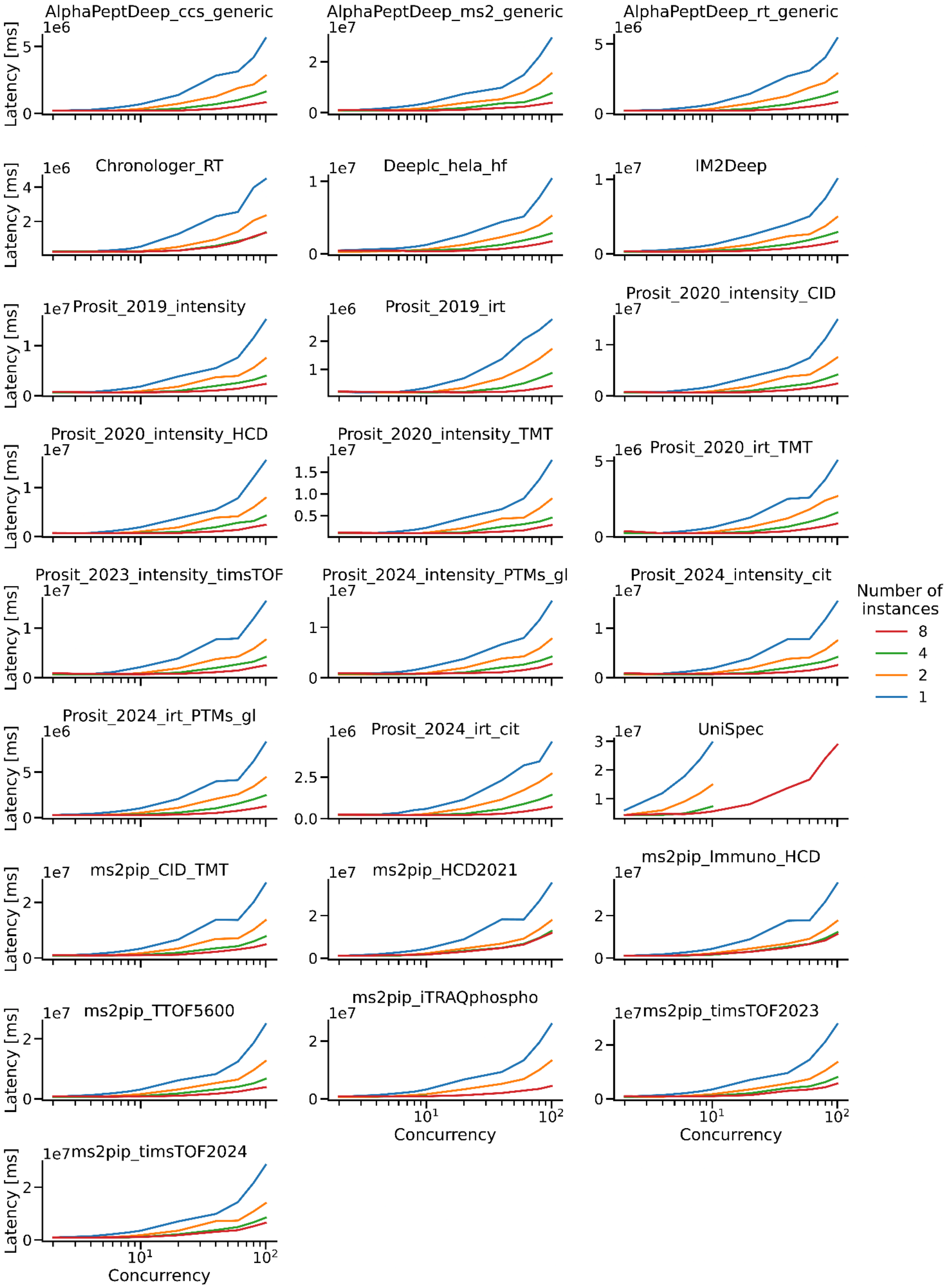
AlphaPeptDeep_ccs_generic
AlphaPeptDeep_ms2_generic
AlphaPeptDeep_rt_generic
Chronologer_RT
Deeplc_hela_hf
IM2Deep
Prosit_2019_intensity
Prosit_2019_irt
Prosit_2020_intensity_CID
Prosit_2020_intensity_HCD
Prosit_2020_intensity_TMT
Prosit_2020_irt_TMT
Prosit_2023_intensity_timsTOF
Prosit_2024_intensity_PTMs_gl
Prosit_2024_intensity_cit
Prosit_2024_irt_PTMs_gl
Prosit_2024_irt_cit
UniSpec
ms2pip_CID_TMT
ms2pip_HCD2021
ms2pip_Immuno_HCD
ms2pip_TTOF5600
ms2pip_iTRAQphospho
ms2pip_timsTOF2023
ms2pip_timsTOF2024
Latency [ms]
Concurrency
Number of instances
8
4
2
1

Supplementary Figure 13

Benchmarking results of models available on Koina measured in median latency in microseconds according to model, client-side concurrency (2,4,6,8,10,20,40,60,80,100), and number of Koina instances used. The client location is Ann Arbor, Michigan, USA server location is Freising, Germany. Peptides used in this Benchmark have a length of 15 amino acids. Source data are provided as a Source Data file.

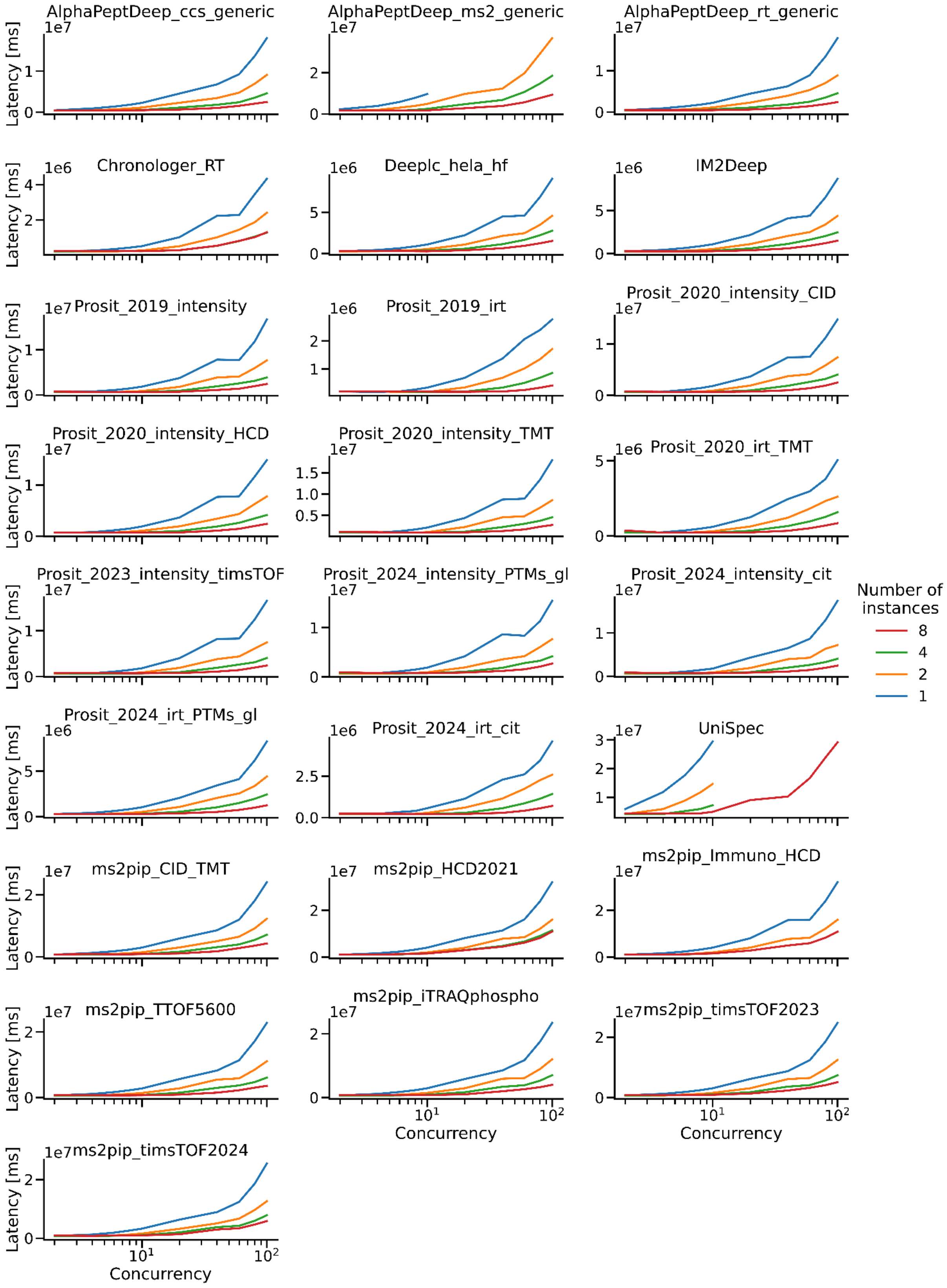
AlphaPeptDeep_ccs_generic
AlphaPeptDeep_ms2_generic
AlphaPeptDeep_rt_generic
Chronologer_RT
Deeplc_hela_hf
IM2Deep
Prosit_2019_intensity
Prosit_2019_irt
Prosit_2020_intensity_CID
Prosit_2020_intensity_HCD
Prosit_2020_intensity_TMT
Prosit_2020_irt_TMT
Prosit_2023_intensity_timsTOF
Prosit_2024_intensity_PTMs_gl
Prosit_2024_intensity_cit
Prosit_2024_irt_PTMs_gl
Prosit_2024_irt_cit
UniSpec
ms2pip_CID_TMT
ms2pip_HCD2021
ms2pip_Immuno_HCD
ms2pip_TTOF5600
ms2pip_iTRAQphospho
ms2pip_timsTOF2023
ms2pip_timsTOF2024
Latency [ms]
Concurrency
Number of instances
8
4
2
1

Supplementary Figure 14

Benchmarking results of models available on Koina measured in median latency in microseconds according to model, client-side concurrency (2,4,6,8,10,20,40,60,80,100), and number of Koina instances used. The client location is Ann Arbor, Michigan, USA server location is Freising, Germany. Peptides used in this Benchmark have a length between 7 and 30 amino acids. Source data are provided as a Source Data file.

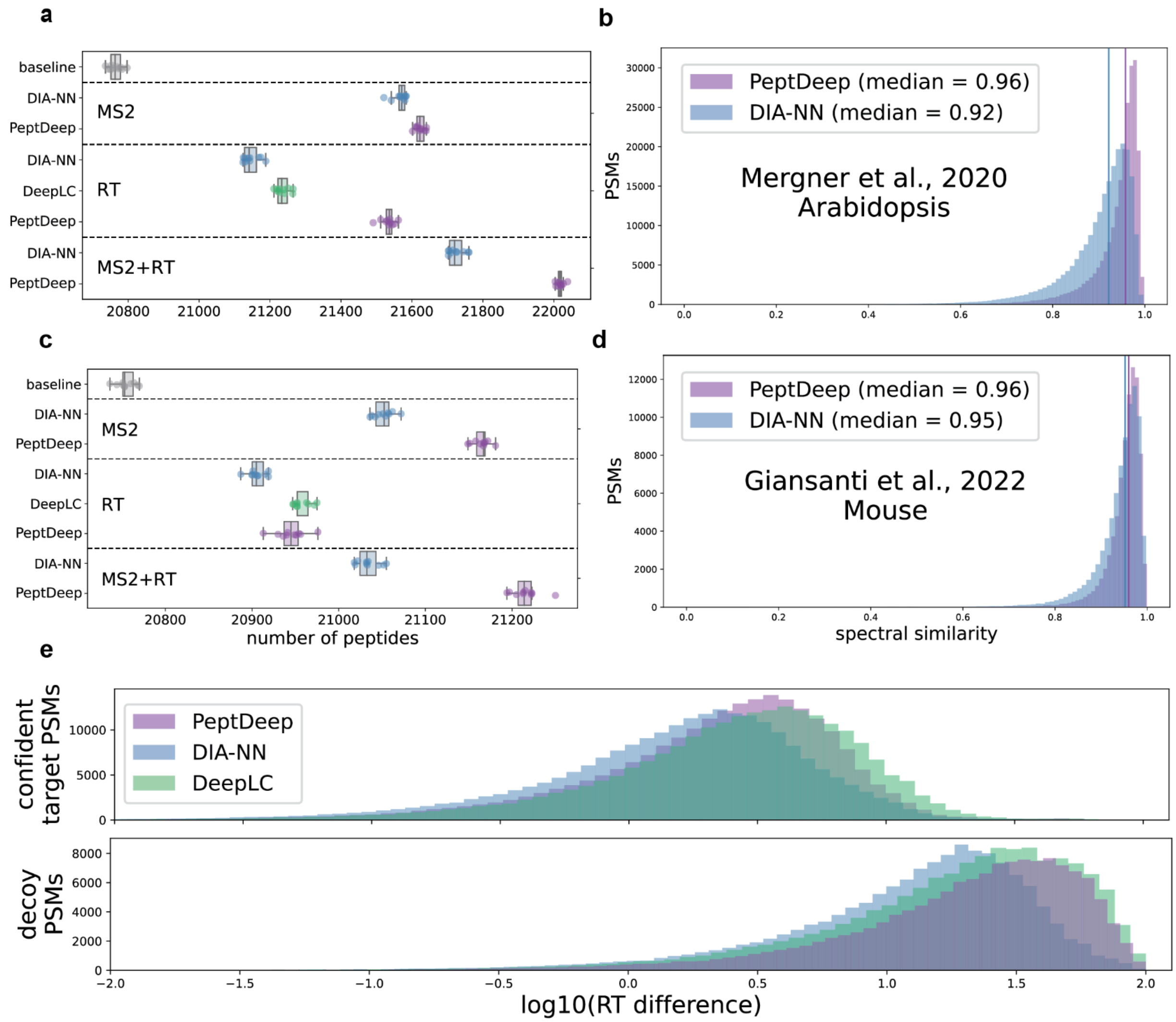

Supplementary Figure 15

Phosphoproteomics rescoring with various models. (a) Numbers of peptides identified using different models on the Arabidopsis thaliana data across 30 tissues[1]. n=10 Percolator runs with different random seeds were performed to estimate the variance in peptide identifications for each model. “Baseline” signifies the peptides identified when excluding MSBooster before Percolator rescoring. MSBooster calculated and added the unweighted spectral entropy feature (MS2), delta RT loess feature (RT), or both (MS2+RT). Boxplots show the interquartile range

(IQR) and median, with whiskers at 1.5 times the IQR below the first quartile and above the third quartile. Swarmplots are plotted on top, and any point outside the boxplot whiskers is considered an outlier. (b) The distribution of spectral similarity scores for confident target PSMs (those PSMs with lower expectation values ("e-values") than the lowest e-value assigned to a decoy PSM in the same pin file). The features were calculated using predictions either from PeptDeep, DeepLC, or DIA-NN. (c-d) The same as (a-b) but for the 8 mouse pancreatic ductal adenocarcinoma cell lines[2]. (e) The distribution of delta RT loess scores for confident target and decoy phosphorylated PSMs from the Arabidopsis thaliana dataset. The log10 of each value plus a small pseudocount was added for clearer visualization of the distribution differences. Source data are provided as a Source Data file.

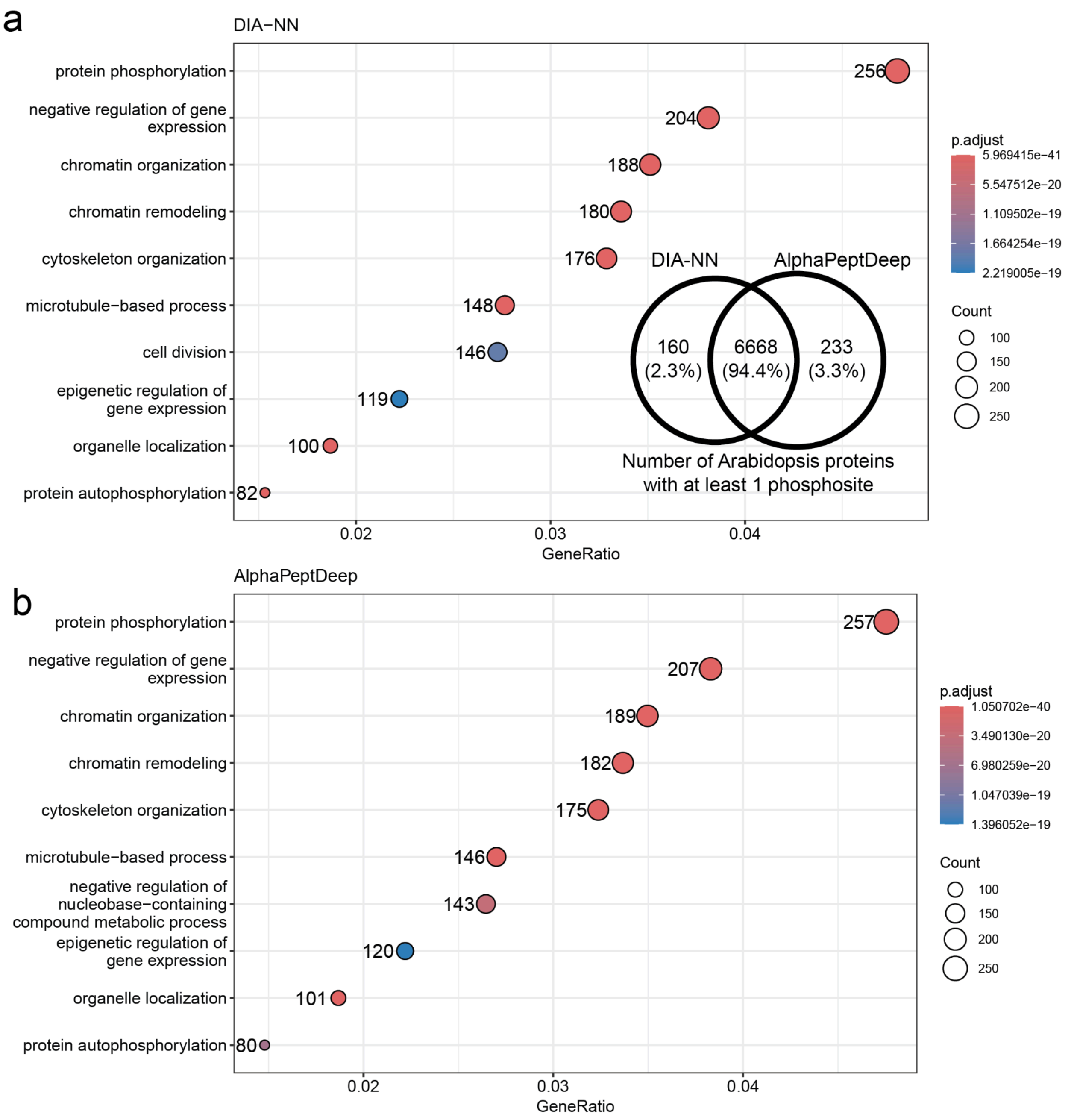

Supplementary Figure 16

Overrepresentation tests for biological processes were performed on final protein lists when using DIA-NN (a) or AlphaPeptDeep (b) for peptide MS2 and retention time prediction. Only proteins with at least 1 phosphosite were considered, and contaminant proteins were filtered out so as to only include Arabidopsis proteins. Each protein list was tested against a background of all Arabidopsis proteins in the Bioconductor OrgDb. Numbers next to each dot represent the number of proteins detected belonging to each biological process. The Venn diagram inset in (a)

shows the overlap between the two models' protein sets. Source data are provided as a Source Data file.

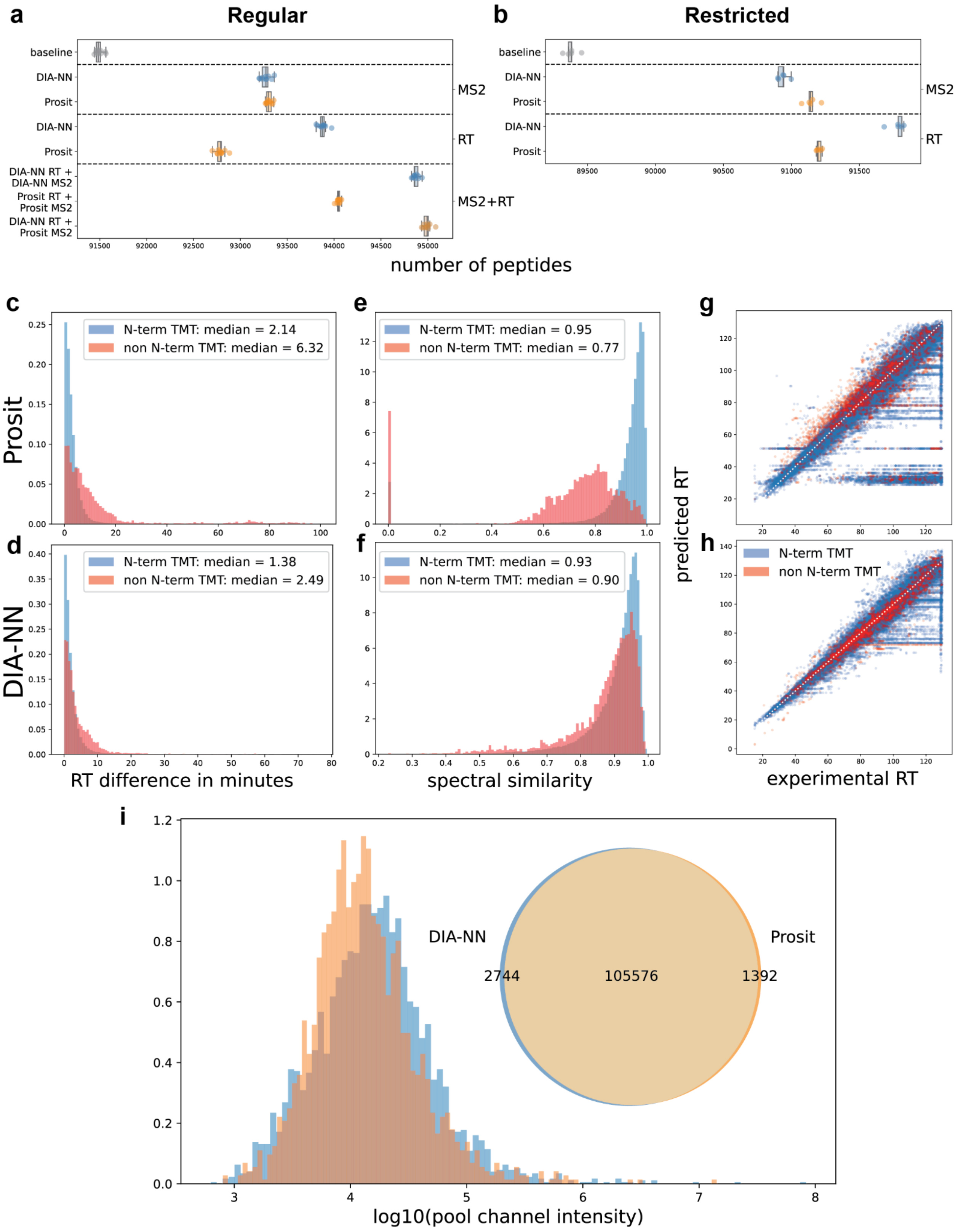

Supplementary Figure 17

Comparison of TMT models for the LUAD dataset[3]. (a) Number of TMT11-labeled peptides identified with 10 iterations of PSM rescoring. (b) The number of TMT peptides identified when the database search is “restricted”. In-silico digest was done only up to 30 amino acids long, and N-terminal TMT was a fixed modification. (c-f) Histograms depicting feature scores for confident target PSMs separated by if they are N-terminally labeled (blue) or not (red). c-d depicts the RT difference score, while e-f depicts the spectral similarity score. (g-h) Scatterplots of confident target PSMs. The x-axis denotes the experimental RT in minutes, while the y-axis shows the predicted RT, after it has been calibrated to the same scale as the experimental RT. Predictions are from Prosit (g) or DIA-NN (h). A white dotted line of the equation x=y shows where a perfect alignment of experimental and predicted RT would lie. (i) 2744 and 1392 unique TMT PSMs were identified by DIA-NN and Prosit. The 126 channel was a pooled sample and non-zero reporter ion intensities from this channel are visualized here. Source data are provided as a Source Data file.

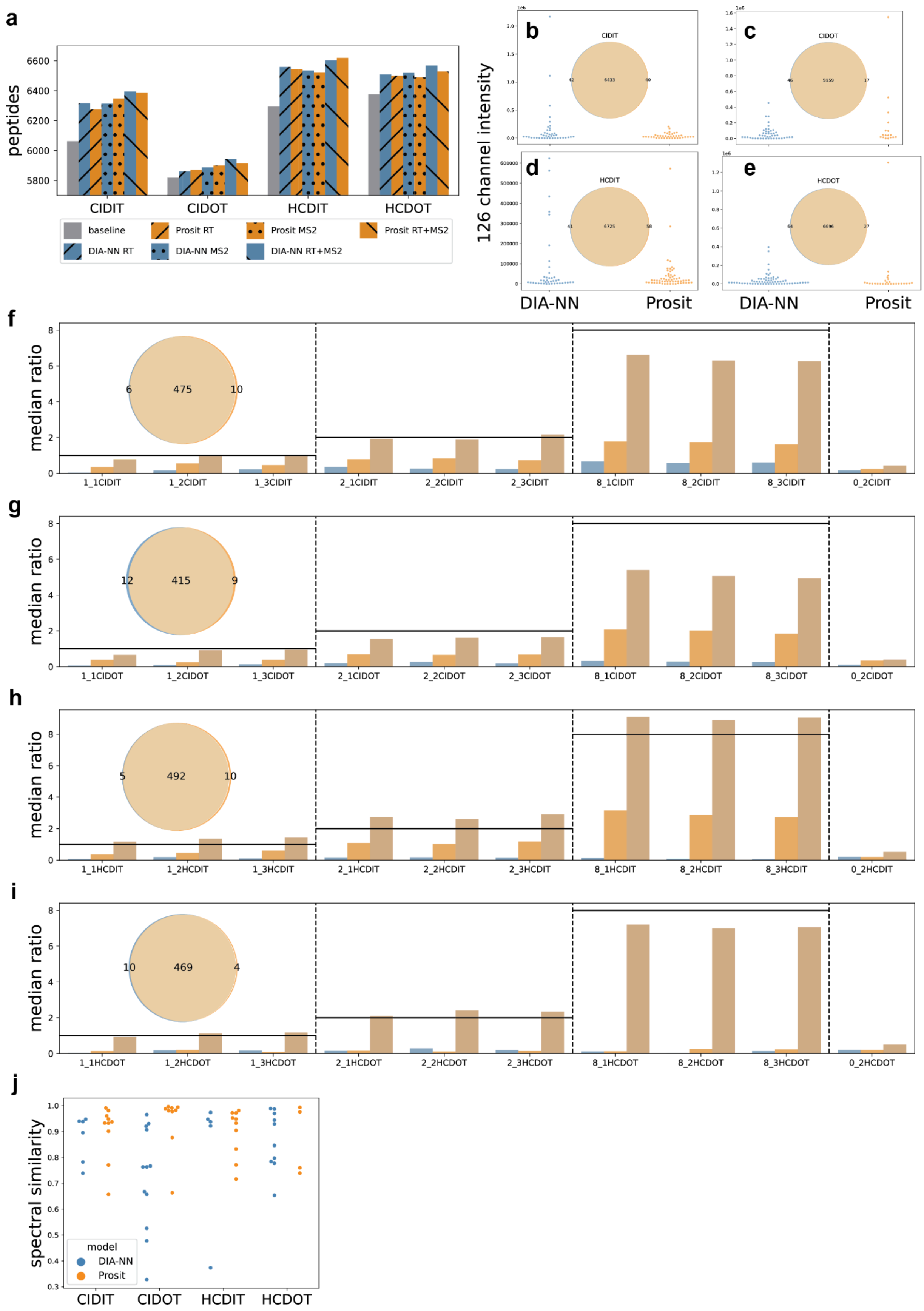
a
peptides
CIDIT
CIDOT
HCDIT
HCDOT
baseline
Prosit RT
Prosit MS2
Prosit RT+MS2
DIA-NN RT
DIA-NN MS2
DIA-NN RT+MS2
b
c
d
e
126 channel intensity
DIA-NN
Prosit
f
g
h
i
median ratio
j
spectral similarity
model
DIA-NN
Prosit

Supplementary Figure 18

(a) TMT11 peptides identified in a yeast/human proteome mixture[4]. 4 combinations of settings were produced by matching CID/HCD fraction with orbitrap (OT) or ion trap (IT) analyzer. Runs using DIA-NN's predictions are in blue and runs using Prosit's are in orange, with separate hatch marks designating whether RT, MS2, or both feature types were used. (b-e) Unique PSMs from each model were selected, and the reporter ion intensities in the 126 channel were visualized in a swarm plot. Both human and yeast PSMs were included here. (f-i) Quantified yeast peptides from DIA-NN and Prosit were compared via a Venn diagram. For each subset of this Venn diagram, the median ratio of the peptides in each channel to the reference 126 TMT channel is visualized, with a horizontal black line indicating what the expected ratio is. (j) For the unique peptides from each model, the highest spectral similarity for PSMs from each peptide is plotted. Source data are provided as a Source Data file.

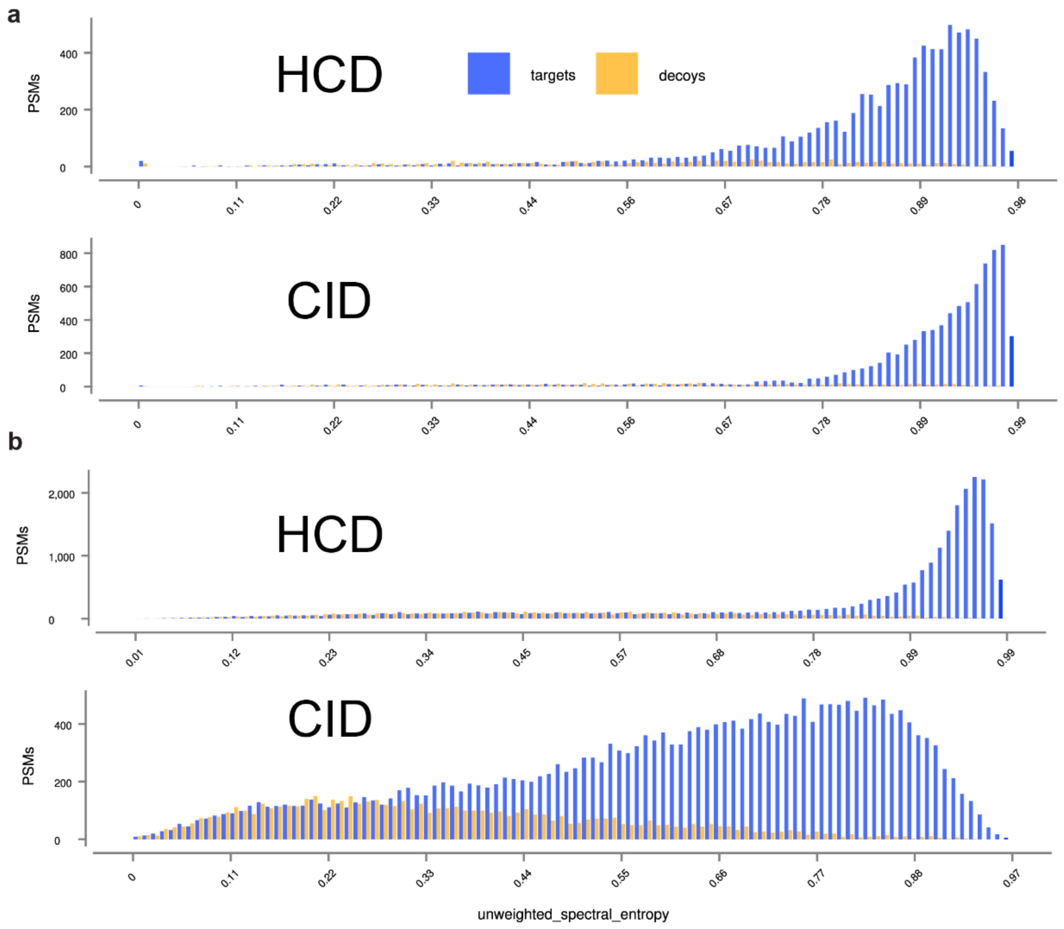

Supplementary Figure 19

Target and decoy PSM distributions for the unweighted spectral entropy MS/MS similarity feature when comparing experimental fragment ion intensities with predicted intensities from the Prosit_2020_intensity HCD and CID models. Distributions are shown for PSMs from one pin file each from a) Marcu et al., 2021[5] and b) Pak et al., 2021[6]. Source data are provided as a Source Data file.

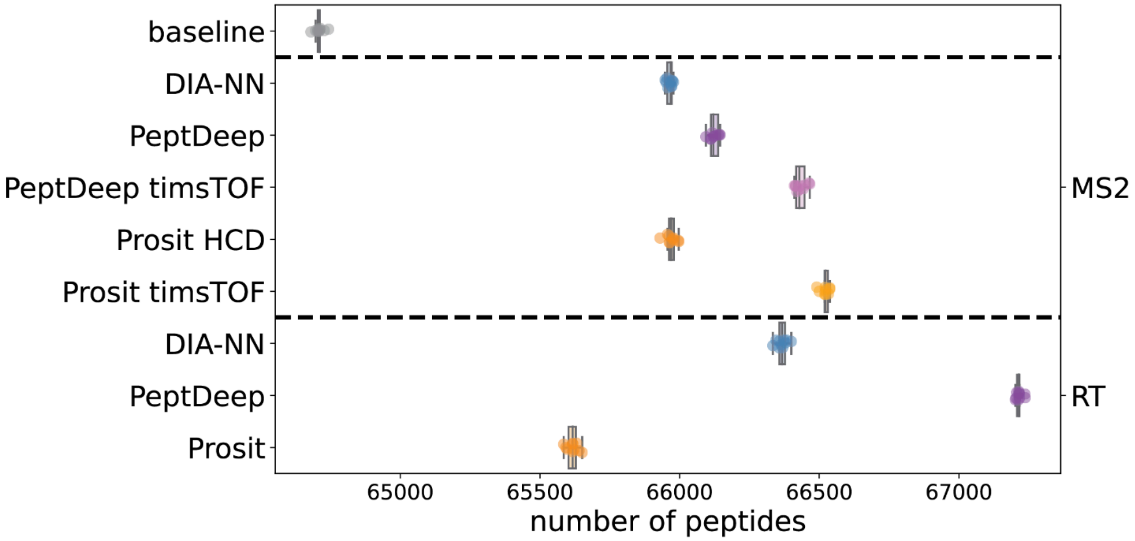

Supplementary Figure 20

Swarmplot and box-and-whisker plot of the peptides identified in data collected on a timsTOF mass spectrometer from Meier et al., 2018[7]. Source data are provided as a Source Data file.

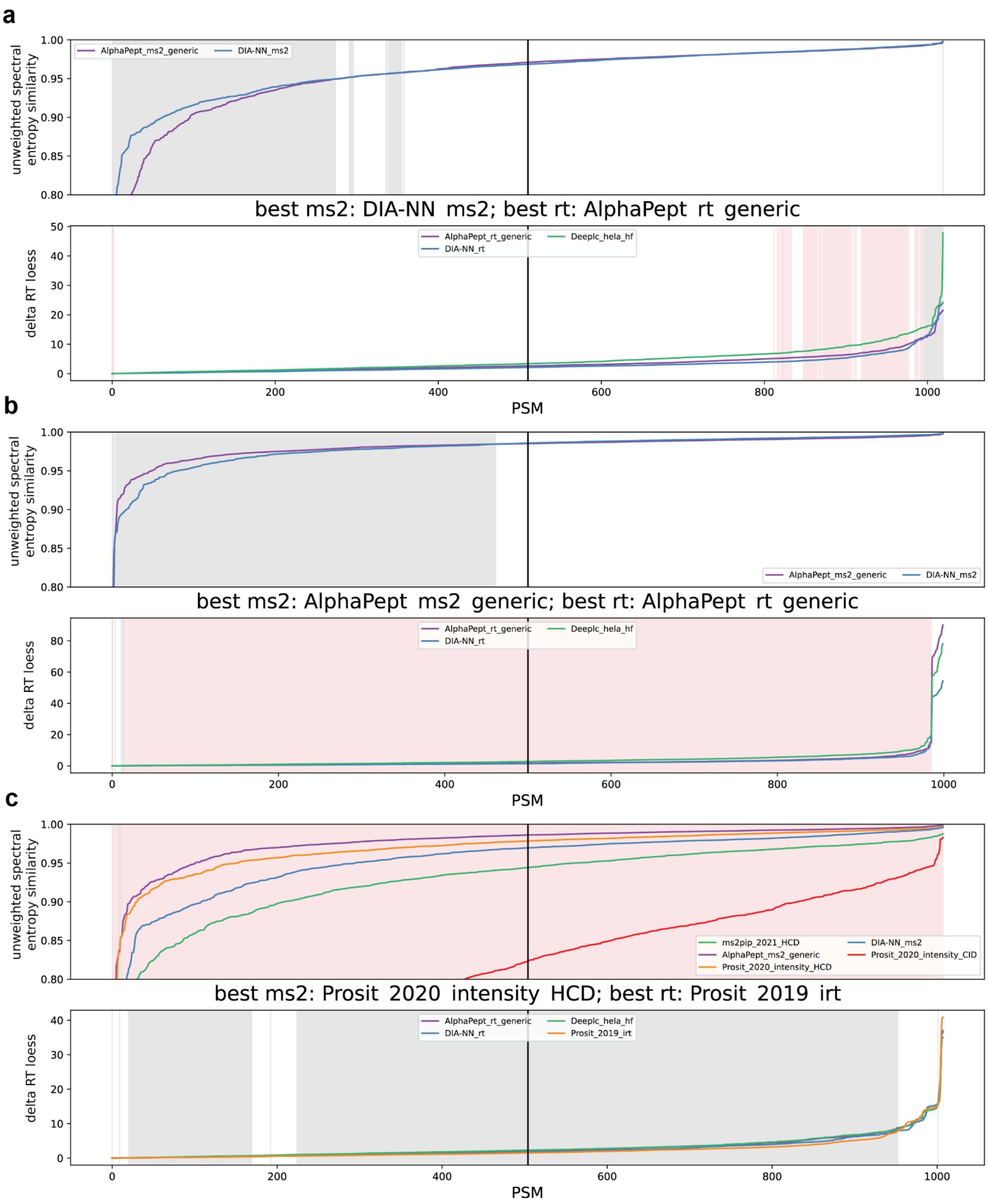
a
unweighted spectral entropy similarity
AlphaPept_ms2_generic
DIA-NN_ms2
best ms2: DIA-NN ms2; best rt: AlphaPept rt generic
delta RT loess
AlphaPept_rt_generic
DIA-NN_rt
Deeplc_hela_hf
PSM
b
unweighted spectral entropy similarity
AlphaPept_ms2_generic
DIA-NN_ms2
best ms2: AlphaPept ms2 generic; best rt: AlphaPept rt generic
delta RT loess
AlphaPept_rt_generic
DIA-NN_rt
Deeplc_hela_hf
PSM
c
unweighted spectral entropy similarity
ms2pip_2021_HCD
AlphaPept_ms2_generic
Prosit_2020_intensity_HCD
DIA-NN_ms2
Prosit_2020_intensity_CID
best ms2: Prosit 2020 intensity HCD; best rt: Prosit 2019 irt
delta RT loess
AlphaPept_rt_generic
DIA-NN_rt
Deeplc_hela_hf
Prosit_2019_irt
PSM

d

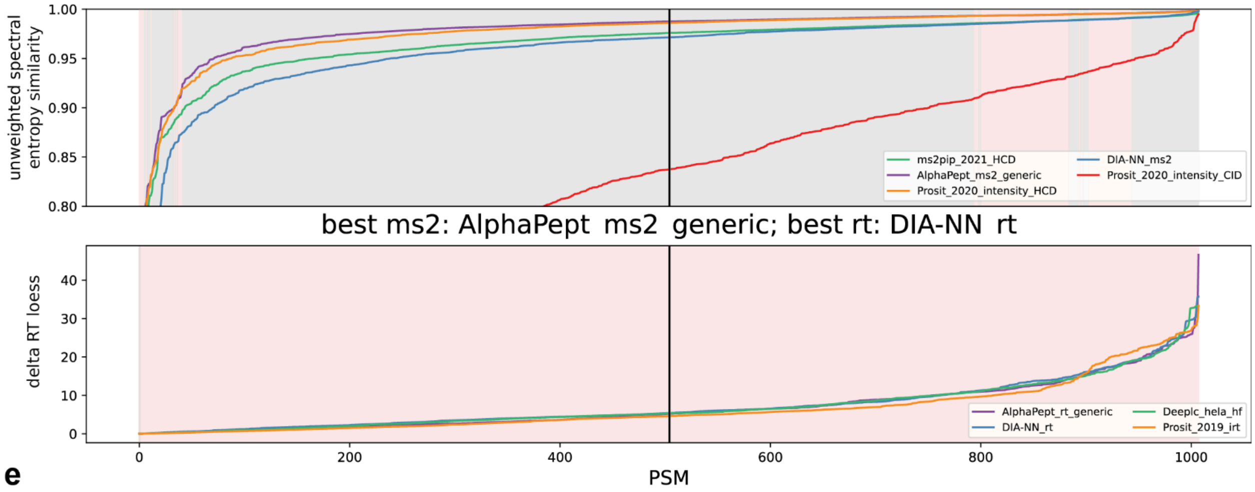

unweighted spectral entropy similarity
ms2pip_2021_HCD
AlphaPept_ms2_generic
Prosit_2020_intensity_HCD
DIA-NN_ms2
Prosit_2020_intensity_CID
best ms2: AlphaPept ms2 generic; best rt: DIA-NN rt
delta RT loess
AlphaPept_rt_generic
DIA-NN_rt
Deeplc_hela_hf
Prosit_2019_irt
PSM

e

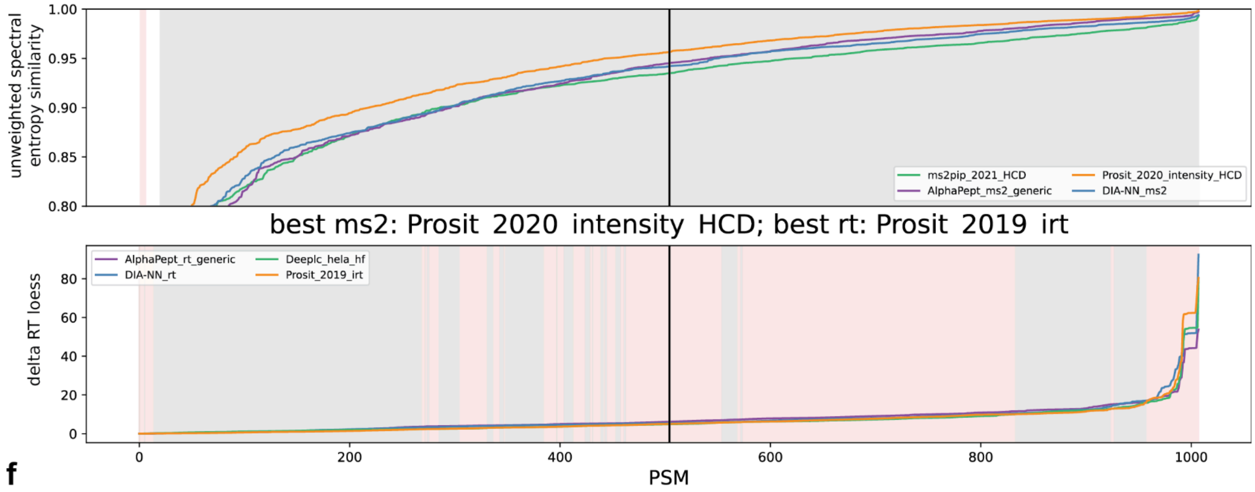

unweighted spectral entropy similarity
ms2pip_2021_HCD
AlphaPept_ms2_generic
Prosit_2020_intensity_HCD
DIA-NN_ms2
best ms2: Prosit 2020 intensity HCD; best rt: Prosit 2019 irt
delta RT loess
AlphaPept_rt_generic
DIA-NN_rt
Deeplc_hela_hf
Prosit_2019_irt
PSM

f

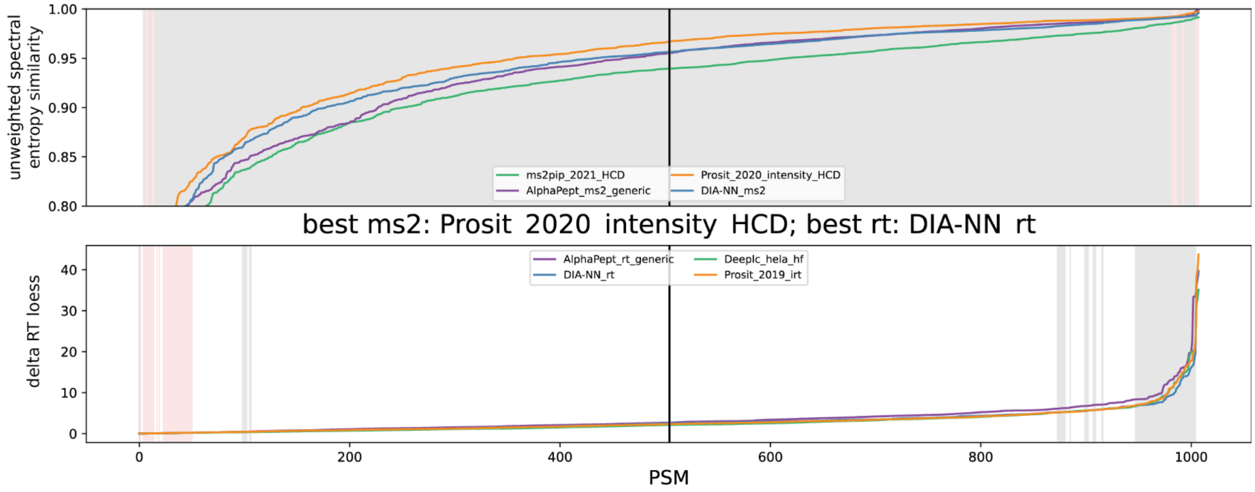

unweighted spectral entropy similarity
ms2pip_2021_HCD
AlphaPept_ms2_generic
Prosit_2020_intensity_HCD
DIA-NN_ms2
best ms2: Prosit 2020 intensity HCD; best rt: DIA-NN rt
delta RT loess
AlphaPept_rt_generic
DIA-NN_rt
Deeplc_hela_hf
Prosit_2019_irt
PSM

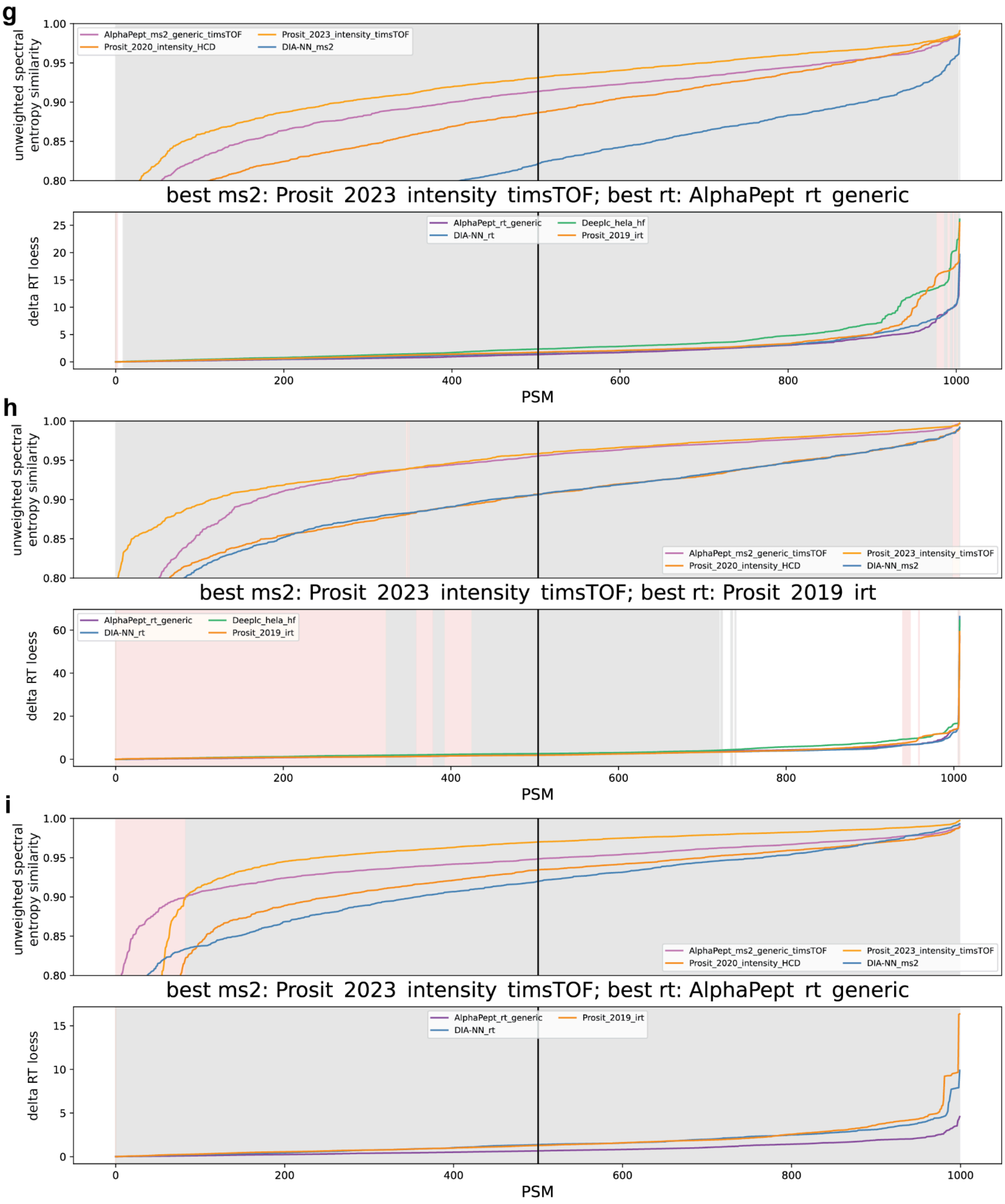
g
unweighted spectral entropy similarity
AlphaPept_ms2_generic_timsTOF
Prosit_2020_intensity_HCD
Prosit_2023_intensity_timsTOF
DIA-NN_ms2
best ms2: Prosit_2023_intensity_timsTOF; best rt: AlphaPept_rt_generic
delta RT loess
AlphaPept_rt_generic
DIA-NN_rt
Deeplc_hela_hf
Prosit_2019_irt
PSM
h
unweighted spectral entropy similarity
AlphaPept_ms2_generic_timsTOF
Prosit_2020_intensity_HCD
Prosit_2023_intensity_timsTOF
DIA-NN_ms2
best ms2: Prosit_2023_intensity_timsTOF; best rt: Prosit_2019_irt
delta RT loess
AlphaPept_rt_generic
DIA-NN_rt
Deeplc_hela_hf
Prosit_2019_irt
PSM
i
unweighted spectral entropy similarity
AlphaPept_ms2_generic_timsTOF
Prosit_2020_intensity_HCD
Prosit_2023_intensity_timsTOF
DIA-NN_ms2
best ms2: Prosit_2023_intensity_timsTOF; best rt: AlphaPept_rt_generic
delta RT loess
AlphaPept_rt_generic
DIA-NN_rt
Prosit_2019_irt
PSM

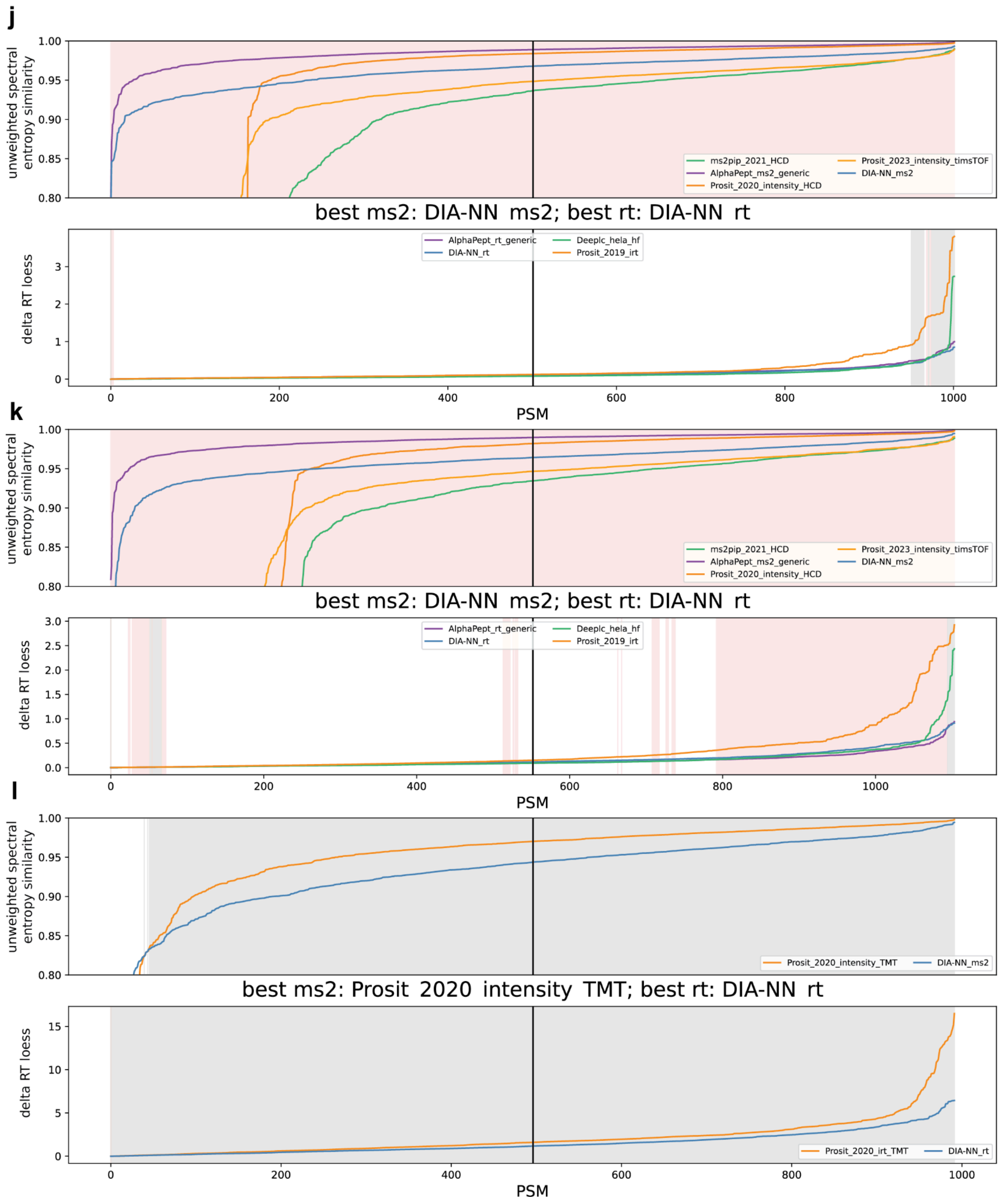

Supplementary Figure 21

Feature values for top PSMs for each model tested during the heuristic model search, just as in Fig 5. The datasets covered here are Arabidopsis phosphoproteome[1] (a), mouse

phosphoproteome[2] (b), HLA DDA class II[5] (c), HLA DDA class I[6] (d), HLA DIA class I from Pak et al., 2021[6] (e) and from Ritz et al., 2017[8] (f), HLA DDA class I and II on timsTOF SCP[9] (g-h), HeLa tryptic digest on timsTOF[7] (i), Astral DIA from Serrano et al., 2024[10] (j) and Guzman et al. 2024[11] (k), and TMT11 LUAD[3] (l). Source data are provided as a Source Data file.

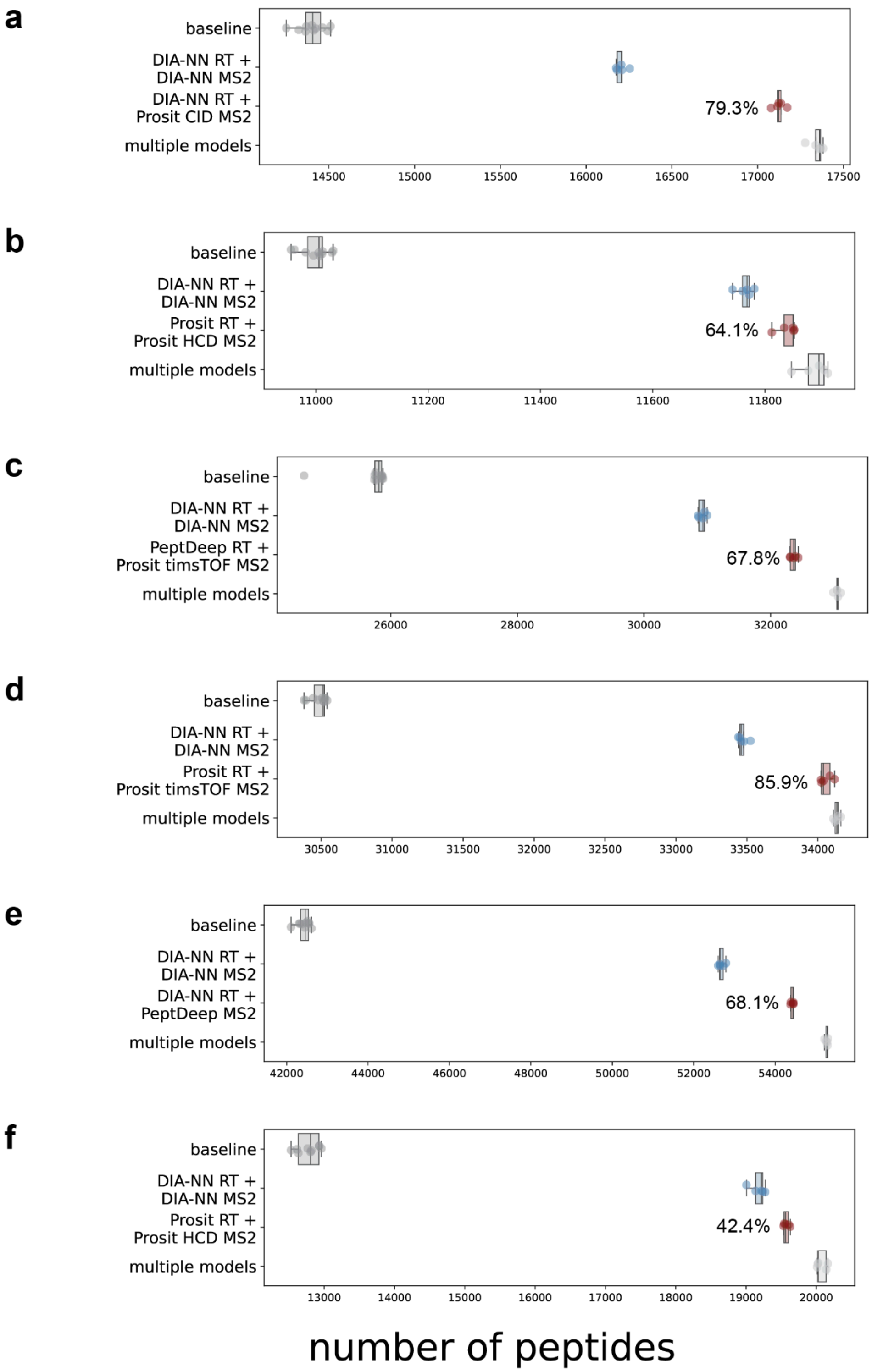

a
baseline
DIA-NN RT +
DIA-NN MS2
DIA-NN RT +
Prosit CID MS2
multiple models
79.3%
14500
15000
15500
16000
16500
17000
17500
b
baseline
DIA-NN RT +
DIA-NN MS2
Prosit RT +
Prosit HCD MS2
multiple models
64.1%
11000
11200
11400
11600
11800
c
baseline
DIA-NN RT +
DIA-NN MS2
PeptDeep RT +
Prosit timsTOF MS2
multiple models
67.8%
26000
28000
30000
32000
d
baseline
DIA-NN RT +
DIA-NN MS2
Prosit RT +
Prosit timsTOF MS2
multiple models
85.9%
30500
31000
31500
32000
32500
33000
33500
34000
e
baseline
DIA-NN RT +
DIA-NN MS2
DIA-NN RT +
PeptDeep MS2
multiple models
68.1%
42000
44000
46000
48000
50000
52000
54000
f
baseline
DIA-NN RT +
DIA-NN MS2
Prosit RT +
Prosit HCD MS2
multiple models
42.4%
13000
14000
15000
16000
17000
18000
19000
20000
number of peptides

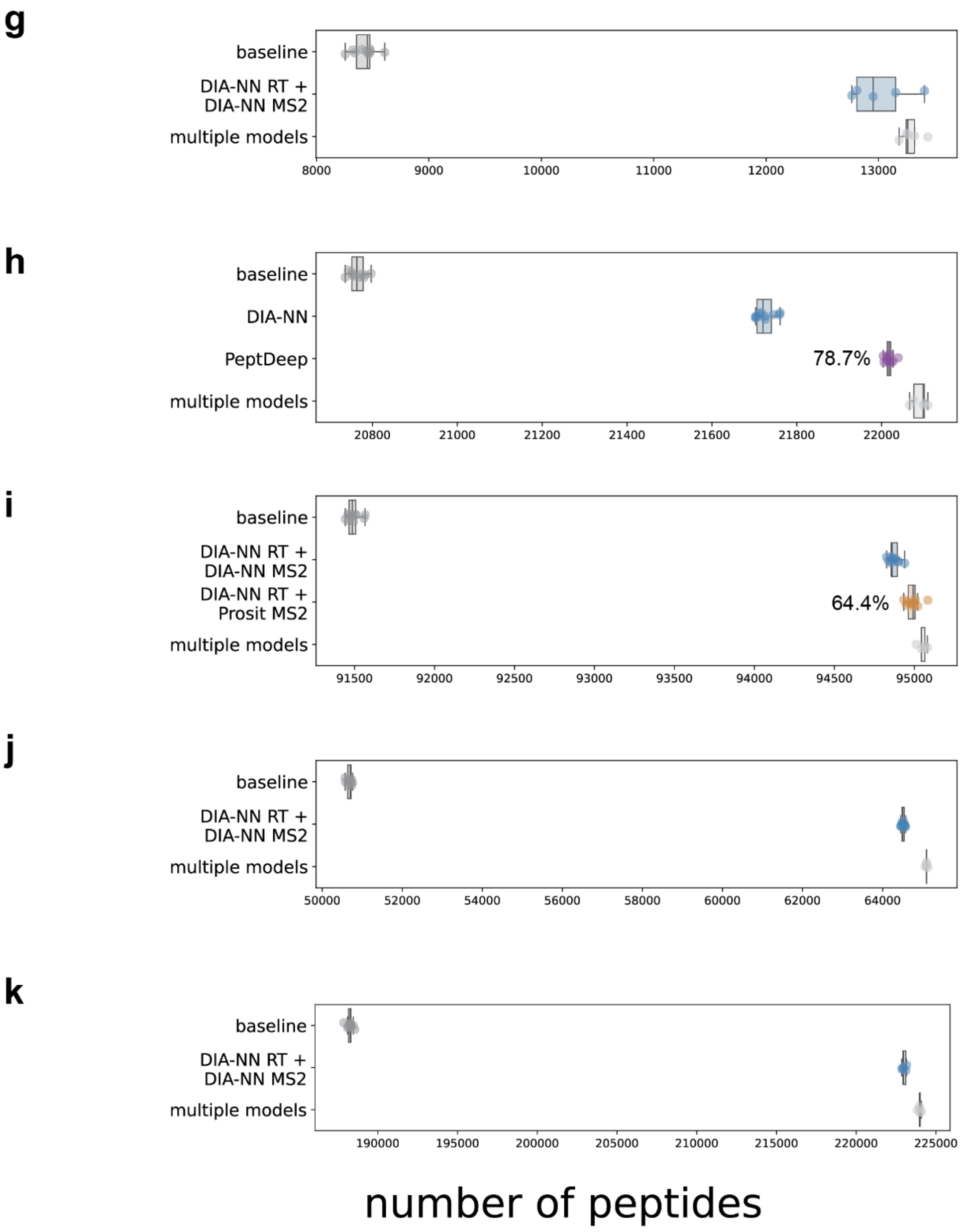

Supplementary Figure 22

The number of peptide identifications using DIA-NN, multiple models, or the best single MS2 and RT models combined, if it outperformed DIA-NN in Supplementary Data 2. HLA DDA class I and II from Marcu et al., 2021[5] (a-b), HLA DDA class I and II on timsTOF SCP[9] (c-d), HLA DDA and DIA class I from Pak et al., 2021[6] (e-f), HLA DIA class I from Ritz et al., 2017[8] (g), Arabidopsis phosphoproteome[1] (h), TMT11 LUAD[3] (i), and Astral DIA from Serrano et al., 2024[10] (j) and Guzman et al. 2024[11] (k). The percentage value was calculated from the median values

of the test conditions: (“best single model combination” - “DIA-NN”) / (“multiple models” - “DIA-NN”). Source data are provided as a Source Data file.

Supplementary Tables

	DIA-NN	Prosit	AlphaPeptDeep	MS2PIP	DeepLC
AA length limit	No	30	No	30	60
Phospho	Yes	No	Yes	No	Yes
n-term TMT optional	Yes	No			

Supplementary Table 1

Models prediction limitations. We consider if long peptides (AA length limit), phosphopeptides (Phospho), and peptides without N-terminal TMT labelling (n-term TMT optional) are allowed. This table does not consider the Prosit PTMs model, which was made available on Koina after the time of writing.

Supplementary Notes

Comparison with existing platforms

BioModelsML

BioModelsML aims to solve a related but ultimately different issue inherent in the publication process of ML models. BioModelsML puts special emphasis on reproducibility & validation of results published in the corresponding paper. This stands in contrast to Koina, which aims to improve the reproducibility of models but not of the results published in the manuscript. BioModelsML provides standalone docker containers that can be used to re-train the model and reproduce published figures, ensuring users can achieve the results claimed in the manuscript. These individual docker containers have unique input/output formats. Interoperability between models is thus very limited in comparison to Koina where interoperability between models is considered of crucial importance. Additionally, BioModelsML is, unlike Koina, not an active service which is one of the foundational pillars that the “democratization” aspect & the integration into third-party tools rely on. Ultimately, Koina and BioModelsML complement each other very well, and they have the potential for future collaboration.

Kipoi

Both Kipoi and Koina aim to democratize machine learning model usage in scientific research, with Kipoi focusing on genomics and offering a programmatic standard and API for accessing an extensive repository of models for tasks like transcription factor binding prediction. In contrast,

Koina is designed explicitly for proteomics, providing web-accessible and locally usable high-performance machine-learning models for predicting peptide properties. A key advantage of Koina is its web-based remote execution of models via HTTP(S) requests, simplifying access for proteomics researchers without requiring extensive computational expertise or local installations. Koina also offers local and remote usage, which is crucial to enable direct and user-friendly integration with proteomics software like FragPipe, Skyline, and EncyclopeDIA. Furthermore, Koina can leverage notable advantages due to its focus on the proteomics domain, such as the common model interface based on ProForma 2.0 and the automated benchmarking and optimal model selection for PSM rescoring. While Kipoi provides programmatic access and supports various genomics applications, Koina's features are more directly tailored to the needs and challenges of the proteomics research community, offering enhanced accessibility and integration of ML models within existing proteomics workflows.

Additional MSBooster PSM rescoring

Introduction

Koina integration in MSBooster enables users to compare model performance systematically by keeping all other data processing parameters the same. Because of differences in model architectures and training data, we hypothesize that the optimal model for rescoring will depend on the specifics of the dataset. To investigate this, we considered various types of data to pinpoint patterns between specific proteomics data types and optimal models. Seven HLA class I/II datasets spanning acquisition modes and instruments were benchmarked in the main text (Figure 4). The benchmarking tests for the remaining dataset are presented here.

Benchmarking tests were performed in Spring 2024, before many of the models currently on Koina were available. Described below are the tests performed to benchmark the available models at that time, which can be used as a framework to compare future models. As MSBooster and FragPipe continue to add support for more models, the heuristic best model search (Figure 5) can quickly estimate if a new model will perform well for PSM rescoring. It is also important to note that the models we find to perform the best in FragPipe may differ from those that are the best in another proteomics software pipeline, attributable to factors such as scoring metrics and learned PSM score discriminator, if Percolator is not used.

Phosphoproteomics

We first considered phosphoproteomics data. Along with DIA-NN, the only currently available Koina models (at the time of writing) that support phosphopeptide prediction are PeptDeep and DeepLC. We benchmarked model performance for two datasets. First, we processed phosphoproteome data from 30 different Arabidopsis thaliana tissues[1] and counted how many phosphopeptides were identified. DIA-NN’s MS2 feature achieved an average improvement of 3.9% over baseline performance (i.e. skipping MSBooster), while PeptDeep’s MS2 feature had a 4.1% improvement (Supplementary Figure 15a). PeptDeep’s improved MS/MS predictions are also evident in Supplementary Figure 15b, where its predictions for high-scoring “confident target” PSMs are concentrated closer to the maximum similarity of 1. The RT feature tells a

similar story (DIA-NN +1.8%, DeepLC +2.3%, PeptDeep +3.7%) (Supplementary Figure 15a). It is less apparent from the distributions of the RT feature which model performs best (Supplementary Figure 15e). DIA-NN's RT predictions improved identifications the least in the Arabidopsis dataset, yet its confident target PSMs have the smallest RT differences. However, DIA-NN also assigned decoy PSMs smaller RT differences compared to the other models, which decreased target-decoy separation and ultimately led Percolator to find the RT feature less informative. Using the RT and MS2 features together, we achieved a 4.6% increase over baseline using DIA-NN and 6.0% increase using PeptDeep (Supplementary Figure 15a). This is in line with the magnitude of improvement expected for a closed MSFragger search on tryptic DDA data[12].

Comparison of the three models on 8 mouse pancreatic ductal adenocarcinoma (mPDAC) cell lines[2] showed similar results (Supplementary Figure 15c-d). Interestingly, DeepLC performed similarly to PeptDeep here, identifying 20958 and 20945 phosphopeptides on average, respectively. Based on these findings, we believe that PeptDeep should be used instead of DIA-NN for phosphoproteomics analysis.

To further illustrate the benefit of using PeptDeep over DIA-NN in phosphoproteomics analyses, we performed overrepresentation tests on the two models' different phosphoprotein lists from the Arabidopsis sample (Supplementary Data 3). We first noted that the majority of the proteins with at least 1 phosphosite were the same when using the two models, with only 2.3% and 3.3% of the phosphoproteins being unique to DIA-NN and PeptDeep, respectively (Supplementary Figure 16a). When considering overrepresentation of the proteins in biological processes when compared to a background set of all Arabidopsis proteins, we found that most of the 10 largest overrepresented sets were in common between the models (Supplementary Fig 16a-b); this is expected and shows that both models accurately predicted phosphopeptide properties. PeptDeep often found a few more proteins in each of these overrepresented biological processes (e.g. 204 vs 207 proteins involved in "negative regulation of gene expression"). While substituting PeptDeep for DIA-NN did not change the fundamental interpretation of our dataset, it did ultimately identify more proteins that further fill out the protein sets we may be interested in profiling.

Astral DIA

Recently, the Orbitrap Astral mass spectrometer (Thermo Scientific) has piqued the interest of the proteomics community for its wide dynamic range, sensitivity of detecting low abundance precursors, and accurate and precise quantification[13,14]. Its higher acquisition rates compared to Orbitrap analyzers allow for narrower isolation windows, producing spectra of reduced complexity in data independent acquisition (DIA) without sacrificing throughput. With the rising interest in narrow window DIA (nDIA), we analyzed two short LC-gradient datasets using 2 Th isolation windows with an MSFragger-DIA-based workflow in FragPipe (see Main Methods). The first dataset included three technical replicate injections of Hap1 human cell lysate[10]. As there are currently no Astral-specific models on Koina, we investigated whether Orbitrap or timsTOF models showed better performance. We found that for both Prosit and PeptDeep, the Orbitrap

mode performed better on this dataset, though only by a small margin (Prosit: 60392 vs 60169; PeptDeep: 60586 vs 59716) (Supplementary Data 2). Regardless, DIA-NN, which is instrument-agnostic, performed the best both for MS2 and RT features. DeepLC identified fewer peptides than average compared to DIA-NN's RT module, though the difference was insignificant (57645 vs 57673 peptides; two-sided t-test, $p>0.05$). DIA-NN's MS2 and RT features also performed the best when combined. They achieved a 27.3% increase in peptide identifications over baseline. Meanwhile, combining the next best features of DeepLC for RT and PeptDeep for MS/MS only achieved a 25.5% increase over baseline. Similar findings were obtained from the second dataset of 46 fractionations in technical triplicates of HEK293 cells[11]. Here, using DIA-NN for both features increased peptides identified by 18.5% and outperformed PeptDeep MS2 and DeepLC RT combined (17.9%). This lower percent increase in identifications may be attributed to the fractionated dataset's already deeper proteome coverage at baseline, identifying more than 3 times the number of peptides as the first dataset. From these analyses, we find that Astral nDIA experiments may benefit most simply from DIA-NN predictions, though this leaves room for improvement if an Astral-specific model is trained.

TMT

At the time of writing, the only two models available in FragPipe for TMT-based prediction are DIA-NN and Prosit, both of which were trained on hundreds of thousands of sequences[4]. We examined two datasets to see whether these models differed not only in the number of peptides identified, but also in their quantification quality. We first analyzed 36 fractions of one plex of TMT11-labeled peptides from a study of lung adenocarcinoma (LUAD) tumors[3]. On this dataset, Prosit outperformed DIA-NN's MS/MS predictions, but not by a significant amount (93306 vs 93269 TMT-labeled peptides, t-test: $p>0.05$) (Supplementary Figure 17a). Meanwhile, DIA-NN greatly outperformed Prosit's RT predictions (93877 vs 92787, t-test: $p<0.05$). Using both DIA-NN features improves over baseline by 3.7%, in contrast to the 2.8% improvement with Prosit. Using DIA-NN for RT and Prosit for MS2 prediction, we identified 94991 peptides on average, a 3.8% improvement over baseline and significantly more than using DIA-NN to predict both peptide properties (t-test: $p<0.01$). Combining the DIA-NN and Prosit models is enabled by MSBooster's access to DIA-NN and Koina.

DIA-NN is more flexible than Prosit in that it does not assume peptides are TMT-labeled at the N-terminus (Supplementary Table 1). To adapt Prosit predictions to accommodate peptides lacking N-terminal TMT, we simply assign them the RTs of the same peptide sequences but with N-terminal TMT and shift the m/z of their b-ions by the TMT label's mass while retaining the same intensities. Neither model considers over-labeling of TMT on serine, so MSBooster applies the strategy above to create predictions for TMT-serine peptides for both models. To explore how well this strategy works for peptides lacking N-terminal TMT, we compared MSBooster feature scores between peptides without and without the N-terminal label in a subset of high-ranking confident target PSMs, 2.3% of which were unlabeled at the N-terminus. We found that while both DIA-NN and Prosit exhibit higher accuracy for N-terminally labeled peptides, Prosit exhibited more noticeable differences between the two groups both in terms of the RT difference and MS2 similarity features (Supplementary Figure 17c-h). In addition, Prosit

does not support prediction for peptides longer than length 30. MSBooster assigns spectral similarity values of 0 and predicted indexed RTs (iRTs) of 0 to PSMs without peptide predictions, further penalizing Prosit's performance; this is most clearly seen in Supplementary Figure 17d with a large amount of confident target PSMs having spectral similarities of 0, and in Supplementary Figure 17g with the horizontal line (around 55 calibrated predicted RT units) of PSMs straying far from the RT calibration curve. In contrast to this "regular" search, we ran a "restricted" database search, setting N-terminal TMT as a fixed modification and limiting the peptide digest length to 30, to determine if the two models performed more similarly without these biases. Without MSBooster's features, the baseline number of TMT-labeled peptides decreased by more than 2000 (Supplementary Figure 17b). Again, we found that DIA-NN performed best for the RT feature and Prosit for the MS2 feature, though the gap in RT feature performance decreased (Prosit regular vs restricted: 1.4% vs 2.0% increase over baseline; DIA-NN: 2.6% vs 2.7%) and the gap in MS2 feature performance increased (Prosit regular vs restricted: 2.0% vs 2.0%; DIA-NN: 1.9% vs 1.7%). This suggests that when N-terminal TMT labeling is incomplete or when many longer peptides are expected, the flexibility of DIA-NN is beneficial.

When analyzing a separate TMT11-labeled human-yeast protein mixture[4], our initial findings that DIA-NN was best for RT and Prosit for MS/MS did not hold. This dataset had four settings combining either HCD or CID fragmentation with Orbitrap (OT) or ion trap (IT) analyzers. Here we found that DIA-NN and Prosit performed comparably in the number of TMT peptides identified (Supplementary Figure 18a). Interestingly, though Prosit TMT considers the fragmentation mode during prediction and DIA-NN does not (nor was it trained on any CID-fragmented TMT data), the models performed comparably on CID data. N-terminal labeling rates for confident target PSMs were 0.05%, 0.04%, 0.04%, and 0.05% for CID IT, CID OT, HCD IT, and HCD OT, respectively.

We next assessed whether the unique PSMs identified by the two models differed by their reporter ion intensities. In the LUAD dataset, DIA-NN found 2744 unique TMT PSMs to Prosit's 1392 (Supplementary Figure 17i). We compared the pooled channel's MS2-based reporter ion intensities between the unique sets and found a statistically significant difference between their median intensities (DIA-NN: 1.58e4, Prosit: 1.22e4; $p<0.01$, Mann-Whitney U test). However, given the wide dynamic range of reporter ion intensities spanning five orders of magnitude in this dataset, this difference does not seem particularly meaningful. Indeed, none of the four MS3-based acquisition methods in the yeast dataset showed a significant difference between DIA-NN's and Prosit's quantification (Supplementary Figure 18b-e) ($p>0.01$, Mann-Whitney U test).

Finally, we wished to check the validity of the unique peptides from each model. The yeast dataset provides a good benchmark because each channel has a known amount of yeast protein spiked into a constant amount of human protein, resulting in the expected ratios of 1:1:1:2:2:2:8:8:8:0 for yeast proteins in channels 127N to 131C compared to the 126 channel. Therefore, we focused on peptides originating from the yeast proteome to assess quantification accuracy. In the data acquired by CID IT, the two models had 475 quantified yeast peptides in

common (Supplementary Figure 18f). DIA-NN and Prosit had 6 and 10 unique quantified peptides, respectively. The shared, DIA-NN-specific, and Prosit-specific peptide sets all showed increasing median ratios across the triplicate channels, with the shared set most closely following that expected 1:2:8 ratio. The average median ratio for DIA-NN was 0.13, 0.29, and 0.61; for Prosit it was 0.45, 0.78, and 1.71; for the shared set it was 0.93, 1.91, and 6.40. Similar trends exist in the three other acquisition methods (Supplementary Figure 18g-i). Though peptides solely identified by Prosit seemed to exhibit higher median ratios than those from DIA-NN, these differences were not statistically significant ($p>0.01$, Mann-Whitney U test). The unique peptides from both models displayed much lower median ratios than what was expected. While some of these peptides may be false positives, most have high spectral similarities, suggesting that these are real identifications (Supplementary Figure 18j). Potential explanations for these reduced ratios include TMT reporter intensity suppression and insufficient unique peptides in each group making it difficult to achieve an accurate median ratio. Overall, most yeast peptides found by DIA-NN and Prosit were overlapping, and many of the peptides that are unique to each model are likely valid. We do not find sufficient evidence that these unique sets are different in any meaningful way. Therefore, we suggest that these models can be used interchangeably, but DIA-NN should be favored when there are many PSMs matched to peptides that are longer than 30 amino acids or with unlabeled N-termini.

Tutorials

In an effort to further improve the usability of Koina, we created (interactive) tutorials for all methods described in the manuscript. These tutorials will be updated according to future changes to any of these packages.

- EncyclopeDIA
  - https://docs.google.com/document/d/1PffYJEtO6jiQ8adXUBkIHrRpvo3yHoQtmCAzmt0CIHM/edit?tab=t.0
- Oktoberfest
  - https://github.com/wilhelm-lab/oktoberfest/tree/development/tutorials
- MSBooster
  - https://fragpipe.nesvilab.org/docs/tutorial_koina.html
- Skyline
  - https://skyline.ms/wiki/home/software/Skyline/page.view?name=Build%20Koina%20Library
- KoinaPy
  - https://github.com/wilhelm-lab/koina/blob/main/clients/python/test/notebooks/koinapy.ipynb
- KoinaR
  - https://bioconductor.org/packages/release/bioc/vignettes/koinar/inst/doc/koina.html

To ensure long-term availability, we also provide the current version of tutorials as a Zenodo release (https://zenodo.org/records/15077518).

References

1. Mergner, J. *et al.* Mass-spectrometry-based draft of the Arabidopsis proteome. *Nature* **579**, 409–414 (2020).
2. Giansanti, P. *et al.* Mass spectrometry-based draft of the mouse proteome. *Nat. Methods* **19**, 803–811 (2022).
3. Soltis, A. R. *et al.* Proteogenomic analysis of lung adenocarcinoma reveals tumor heterogeneity, survival determinants, and therapeutically relevant pathways. *Cell Rep Med* **3**, 100819 (2022).
4. Gabriel, W. *et al.* Prosit-TMT: Deep Learning Boosts Identification of TMT-Labeled Peptides. *Anal. Chem.* **94**, 7181–7190 (2022).
5. Marcu, A. *et al.* HLA Ligand Atlas: a benign reference of HLA-presented peptides to improve T-cell-based cancer immunotherapy. *J Immunother Cancer* **9**, (2021).
6. Pak, H. *et al.* Sensitive Immunopeptidomics by Leveraging Available Large-Scale Multi-HLA Spectral Libraries, Data-Independent Acquisition, and MS/MS Prediction. *Mol. Cell. Proteomics* **20**, 100080 (2021).
7. Meier, F. *et al.* Online Parallel Accumulation-Serial Fragmentation (PASEF) with a Novel Trapped Ion Mobility Mass Spectrometer. *Mol. Cell. Proteomics* **17**, 2534–2545 (2018).
8. Ritz, D., Kinzi, J., Neri, D. & Fugmann, T. Data-Independent Acquisition of HLA Class I Peptidomes on the Q Exactive Mass Spectrometer Platform. *Proteomics* **17**, (2017).
9. Phulphagar, K. M. *et al.* Sensitive, High-Throughput HLA-I and HLA-II Immunopeptidomics Using Parallel Accumulation-Serial Fragmentation Mass Spectrometry. *Mol. Cell. Proteomics* **22**, (2023).
10. Serrano, L. R. *et al.* The One Hour Human Proteome. *Mol. Cell. Proteomics* **23**, 100760 (2024).
11. Guzman, U. H. *et al.* Ultra-fast label-free quantification and comprehensive proteome

coverage with narrow-window data-independent acquisition. *Nat. Biotechnol.* 1–12 (2024) doi:10.1038/s41587-023-02099-7.

12. Yang, K. L. *et al.* MSBooster: improving peptide identification rates using deep learning-based features. *Nat. Commun.* **14**, 1–14 (2023).
13. Heil, L. R. *et al.* Evaluating the Performance of the Astral Mass Analyzer for Quantitative Proteomics Using Data-Independent Acquisition. *J. Proteome Res.* **22**, 3290–3300 (2023).
14. Stewart, H. I. *et al.* Parallelized Acquisition of Orbitrap and Astral Analyzers Enables High-Throughput Quantitative Analysis. *Anal. Chem.* **95**, 15656–15664 (2023).
