# Supplementary material for: Koina: Democratizing machine learning for proteomics research
Source: Nat Commun. 2025 Nov 11;16:9933. doi: 10.1038/s41467-025-64870-5 (PMC12606132; doi:10.1038/s41467-025-64870-5)
Supplement: Supplementary file 2 — Description of Additional Supplementary Files [file 41467_2025_64870_MOESM2_ESM.pdf]

Description of Additional Supplementary Files

File Name: Supplementary Data 1
Description: Model timing across datasets

File Name: Supplementary Data 2
Description: Peptides identified across datasets and predicted libraries

File Name: Supplementary Data 3
Description: Proteins identified by DIA-NN and AlphaPeptDeep in Arabidopsis phosphoproteomics data
